# Supplementary material for: A randomized trial of safety, acceptability and adherence of three rectal microbicide placebo formulations among young sexual and gender minorities who engage in receptive anal intercourse (MTN-035)
Source: PLoS One. 2023 Apr 12;18(4):e0284339. doi: 10.1371/journal.pone.0284339 (PMC10096248; doi:10.1371/journal.pone.0284339)
Supplement: S1 File — (PDF) [file pone.0284339.s002.pdf]

MTN-035

Acceptability, Tolerability, and Adherence of Three Rectal Microbicide Placebo Formulations among HIV Seronegative Cisgender Men, Transgender Men and Transgender Women Who Engage in Receptive Anal Intercourse

Microbicide Trials Network

Funding Agencies:
Division of AIDS, US National Institute of Allergy and Infectious Diseases
US *Eunice Kennedy Shriver* National Institute of
Child Health and Human Development
US National Institute of Mental Health
US National Institutes of Health

Grant Numbers:
UM1AI068633, UM1AI068615, UM1AI106707

DAIDS Protocol ID: 38459

A Non-IND Study

Protocol Chair: José A. Bauermeister, PhD, MPH

Version 1.0

June 15, 2018

MTN-035

Acceptability, Tolerability, and Adherence of Three Rectal Microbicide Placebo Formulations among HIV Seronegative Cisgender Men, Transgender Men and Transgender Women Who Engage in Receptive Anal Intercourse

TABLE OF CONTENTS

LIST OF ABBREVIATIONS AND ACRONYMS 5
PROTOCOL TEAM ROSTER 8
INVESTIGATOR SIGNATURE FORM 18
PROTOCOL SUMMARY 19
1 KEY ROLES 21
1.1 Protocol Identification 21
1.2 Funding Agencies and Sponsor Identification 22
1.3 Medical Officer 22
1.4 Clinical Laboratories 22
1.5 Data Centers 22
1.6 Study Implementation 23
2 INTRODUCTION 24
2.1 Background of Rectal Microbicide Research and Study Rationale 24
2.2 Description of Study Products 28
2.3 Acceptability of non-gel rectal microbicide formulations 31
2.4 Practices commonly associated with RAI 33
2.5 Study Hypotheses and Rationale for Study Design 34
3 OBJECTIVES 35
3.1 Primary Objectives 35
3.2 Exploratory Objective 35
4 STUDY DESIGN 35
4.1 Identification of Study Design 35
4.2 Summary of Major Endpoints 36
4.3 Description of Study Population 36
4.4 Time to Complete Accrual 36
4.5 Study Groups 36
4.6 Expected Duration of Participation 36
4.7 Sites 37
5 STUDY POPULATION 37
5.1 Selection of the Study Population 37
5.2 Inclusion Criteria 37
5.3 Exclusion Criteria 38
5.4 Co-enrollment Guidelines 39
6 STUDY PRODUCT 40
6.1 Regimen 40
6.2 Administration 40
6.3 Study Product Formulation 41

6.4 Study Product Supply and Accountability 42
6.5 Study Product Dispensing 42
6.6 Ancillary Study Supplies 43
6.7 Concomitant Medications and Practices 43
7 STUDY PROCEDURES 43
7.1 Pre-screening 44
7.2 Screening 44
7.3 Enrollment (Day 0) 46
7.4 Follow-up Visits 47
7.5 Follow-up Procedures for Participants Who Permanently Discontinue Study Product 50
7.6 Protocol Counseling: Adherence and Study Product Use Counseling 51
7.7 Clinical Evaluations and Procedures 51
7.8 Behavioral Evaluations 52
7.9 Laboratory Evaluations 53
7.10 Specimen Management 54
7.11 DAIDS Laboratory Oversight 54
7.12 Biohazard Containment 55
8 ASSESSMENT OF SAFETY 55
8.1 Safety Monitoring 55
8.2 Clinical Data and Safety Review 55
8.3 Adverse Events Definitions and Reporting Requirements 56
8.4 Pregnancy and Infant Outcomes 58
8.5 Regulatory Requirements 58
8.6 Social Harms Reporting 58
9 CLINICAL MANAGEMENT 58
9.1 Grading System 59
9.2 Dose Modification Instructions 59
9.3 General Criteria for Permanent Discontinuation of Study Product 59
9.4 Follow-up in Response to Observed Adverse Events 60
9.5 Sexually Transmitted Infection/Reproductive Tract Infection 60
9.6 HIV-1/2 Infection 60
9.7 Pregnancy 60
9.8 Criteria for Early Termination of Study Participation other than HIV-1/2 Infection and Pregnancy 61
10 STATISTICAL CONSIDERATIONS 61
10.1 Overview and General Design 61
10.2 Study Endpoints 61
10.3 Sample Size and Power 62
10.4 Randomization Procedures 63
10.5 Participant Accrual and Retention 63
10.6 Data and Safety Monitoring Procedures 63
10.7 Data Analyses 64
10.8 Missing Data 64
11 DATA HANDLING AND RECORDKEEPING 65
11.1 Data Management Responsibilities 65

11.2 Source Documents and Access to Source Data/Documents 65
11.3 Quality Control and Quality Assurance 66
12 CLINICAL SITE OVERSIGHT 66
13 HUMAN SUBJECTS PROTECTIONS 66
13.1 Institutional Review Boards/Ethics Committees 66
13.2 Protocol Registration 67
13.3 Study Coordination 68
13.4 Risk Benefit Statement 68
13.5 Informed Consent Process 70
13.6 Participant Confidentiality 70
13.7 Special Populations 71
13.8 Compensation 72
13.9 Communicable Disease Reporting 72
13.10 Access to HIV-related Care 72
13.11 Study Discontinuation 72
14 PUBLICATION POLICY 73
15 APPENDICES 74
APPENDIX I: SCHEDULE OF STUDY VISITS AND EVALUATIONS 75
APPENDIX II: ALGORITHM FOR HIV TESTING FOR SCREENING AND FOLLOW-UP .77
APPENDIX III: SAMPLE INFORMED CONSENT FORM (SCREENING AND ENROLLMENT) 78
REFERENCES 92

TABLE OF FIGURES

Table 1: MTN-035 Study Product Regimen 20
Table 2: Witepsol® H5-based suppository products currently on the market 31
Table 3: Study Product Regimen 40
Table 4: Visit 1- Screening Visit 45
Table 5: Visit 2- Enrollment Visit 46
Table 6: Visits 3, 5 and 7 – First, Second and Third PUEV 47
Table 7: Visits 4 and 6 – Product Switch Visits 48
Table 8: Visit 8 – Final/Early Termination Visit 49
Table 9: Exact 2-sided 95% Confidence Intervals Based on Various Rates of Acceptability or Adherence Endpoints 63
Table 10: Analysis of Safety Event Frequency 63

Figure 1: MTN-035 Study Visit Schedule 20
Figure 2: MTN-035 Study Visit Schedule 43

MTN-035

Acceptability, Tolerability, and Adherence of Three Rectal Microbicide Placebo Formulations among HIV Seronegative Cisgender Men, Transgender Men and Transgender Women Who Engage in Receptive Anal Intercourse

LIST OF ABBREVIATIONS AND ACRONYMS

AE	adverse event
AIDS	Acquired Immunodeficiency Syndrome
AI	anal intercourse
AUC	area under the curve
BRWG	Behavioral Research Working Group
BV	bacterial vaginosis
CASI	computer assisted self-interview
CBC	complete blood count
CDC	Centers for Disease Control and Prevention
CFR	Code of Federal Regulations
CI	Confidence Interval
CRF	case report form
CRS	Clinical Research Site
CT	*Chlamydia trachomatis,* Chlamydia
CTA	Clinical Trial Agreement
CWG	Community Working Group
DAIDS	Division of AIDS
DAIDS PRO	DAIDS Protocol Registration Office
DNA	deoxyribonucleic acid
DOD	directly observed dosing
ENR	enrollment
FDA	(US) Food and Drug Administration
FHCRC	Fred Hutchinson Cancer Research Center
g	grams
GC	*Neisseria gonorrhoeae,* gonorrhea
GCP	Good Clinical Practices
GMP	Good Manufacturing Practices
HCG	human chorionic gonadotropin
HEENT	Head, Eye, Ear, Nose and Throat Examination
HHS	(U.S.) Department of Health and Human Services
HIV	Human Immunodeficiency Virus
HIV-1	Human Immunodeficiency Virus type 1
HSV	herpes simplex virus
HSV-1/2	herpes simplex virus type 1/2
IATA	International Air Transport Association
IB	Investigator's Brochure
ICF	informed consent form
ICH	International Council for Harmonisation

IDI	In-Depth Interview
IEC	Independent Ethics Committee
IM	instant message
IND	Investigational New Drug
IoR	Investigator of Record
IRB	Institutional Review Board
IUD	intrauterine device
IVR	intravaginal ring
kg	kilogram
LC	Laboratory Center
LDMS	Laboratory Data Management System
LOC	Leadership and Operations Center
μg	microgram
MDP	Microbicides Development Programme
mg	milligram
mL	milliliter
mM	millimolar
MO	Medical Officer
mOsm	milliosmole
MSM	men who have sex with men
MTN	Microbicide Trials Network
n	number
N-9	nonoxynol-9
NAAT	nucleic acid amplification test
NF	National Formulary
ng	nanogram
nM	nanomolar
NIAID	National Institute of Allergy and Infectious Diseases
NICHD	National Institute of Child Health and Human Development
NIH	National Institutes of Health
NIMH	National Institute of Mental Health
NL	network laboratory
OHRP	Office for Human Research Protections
PBS	phosphate-buffered saline
PEP	post-exposure prophylaxis
PoR	Pharmacist of Record
PrEP	pre-exposure prophylaxis
PRO	Protocol Registration Office
PSP	Prevention Sciences Program
PSRT	Protocol Safety Review Team
PTID	participant identification
PUEV	product use end visit
RAI	receptive anal intercourse
RE	Regulatory Entity
RH	Relative Humidity
RNA	ribonucleic acid
RSC	Regulatory Support Center
RTI	reproductive tract infection
SAE	serious adverse event

SCHARP	Statistical Center for HIV/AIDS Research & Prevention
SCR	screening
SDMC	Statistical Data Management Center
SMC	Study Monitoring Committee
SOP	standard operating procedure
SMS	short message service
SSP	study specific procedures
STI	sexually transmitted infection
SUSAR	suspected, unexpected serious adverse reaction
TGM	transgender men
TGW	transgender women
UA	urinalysis
UNAIDS	Joint United Nations Programme on HIV/AIDS
UNGASS	United Nations General Assembly Special Session
UPMC	University of Pittsburgh Medical Center
USA	United States of America
USP	United States Pharmacopoeia
UTI	urinary tract infection
WHO	World Health Organization

MTN-035

Acceptability, Tolerability, and Adherence of Three Rectal Microbicide Placebo Formulations among HIV Seronegative Cisgender Men, Transgender Men and Transgender Women Who Engage in Receptive Anal Intercourse

PROTOCOL TEAM ROSTER

Protocol Chair

Jose A. Bauermeister, MPH, PhD
Protocol Chair
University of Pennsylvania
418 Curie Blvd., Suite 222L
Philadelphia, PA 19104
Phone: 215-898-9993
Email: bjose@upenn.edu

Site Investigators

University of Pittsburgh Clinical Research Site (CRS)

Ken Ho, MD
Site Investigator
UPMC, Keystone Building, Suite 531
Division of Infectious Diseases
3520 Fifth Avenue
Pittsburgh, PA 15213 USA
Phone: 412-383-7178
Fax: 412-383-2900
Email: hok2@upmc.edu

Alabama CRS

Craig Hoesley, MD
Site Investigator
University of Alabama at Birmingham
1530 3rd Avenue South, VH 201
Birmingham, AL 35294 USA
Phone: 205-934-3365
Fax: 205-975-3232
Email: choesley@uab.edu

Bridge HIV CRS

Albert Liu, MD, MPH
Site Investigator
Clinical Research Director
Bridge HIV CRS
San Francisco Department of Public Health
25 Van Ness Avenue, Suite 100
San Francisco, CA 94102 USA
Phone: 415-437-7408
Fax: 415-431-7029
Email: albert.liu@sfdph.org

Blantyre CRS

Taha E. Taha, PhD
CTU PI
Johns Hopkins University
Bloomberg School of Public Health,
615 N. Wolfe Street
Baltimore, MD 21205
Phone: 410-614-5255
Fax: 410-502-0688
Email: ttaha@jhsph.edu

Noel Kayange, MBBS, MMED
Site Investigator
Johns Hopkins University Research Project
Chipatala Avenue
P.O. Box 1131
Blantyre, Malawi
Phone: 265-1875-129
Fax: 265-1870-132
Email: nkayange@jhu.medcol.mw

Wits Reproductive Health and HIV Institute (Wits RHI) CRS

Helen Vera Rees, OBE, MBBChir, MA, DRCOG, DCH
CTU Co-PI
Wits Reproductive Health and HIV Institute (Wits RHI)
22 Esselen Street, Hillbrow
Johannesburg, 2001 South Africa
Phone: 27-11-358-5300
Email: hrees@wrhi.ac.za

Thesla Palanee-Phillips, MMed Sci, MSc, PhD
Site Investigator
Wits Reproductive Health and HIV Institute (Wits RHI)
Research Centre
7 Esselen Street, Hillbrow
Johannesburg, 2038 South Africa
Phone: 27-11-358-5471
Fax: 27-86-554-1093
Email: tpalanee@whri.ac.za

Chiang Mai University (CMU) HIV Prevention CRS

Suwat Chariyalertsak, MD, Dr.PH
Site Investigator
Research Institute for Health Sciences
Chiang Mai University
P.O. Box 80 CMU,
Chiang Mai 50202 Thailand
Phone: 66 53 945055
Fax: 66 53 221849
Email: suwat.c@cmu.ac.th

San Miguel CRS

Pedro Gonzales, MD, MAS
Site Investigator
IMPACTA – San Miguel CRS
Calle Putumayo No.177
San Miguel, 15088, Peru
Phone: 51-1- 562-1600 Ext 628
Fax: 51-1- 562-1600 Ext 128
Email: pgonzales@impactaperu.org

US National Institutes of Health (NIH)

Roberta Black, PhD
Chief, Clinical Microbicide Research Branch
Division of AIDS (DAIDS), National Institute of Allergy and Infectious Diseases (NIAID), National Institutes of Health (NIH) - U.S. Department of Health and Human Services (HHS)
5601 Fishers Lane, Room 8B62, MSC 9831
Rockville, MD 20852 USA
Phone: 301-496-8199
Email: rblack@niaid.nih.gov

Naana Cleland, MHCA
Health Specialist, Clinical Microbicide Research Branch (CMRB)
Prevention Sciences Program (PSP) DAIDS, NIAID
NIH – U.S. DHHS
5601 Fishers Lane, Room 8B27, MSC 9831
Rockville, MD 20852 USA
Phone: 240-292-4779
Email: clelandn@niaid.nih.gov

Jeanna Piper, MD
DAIDS Senior Medical Officer
DAIDS/NIAID/NIH/HHS
5601 Fishers Lane, Room 8B68, MSC 9831
Rockville, MD 20852 USA
Phone: 240-292-4798
Email: piperj@niaid.nih.gov

Dianne M. Rausch, PhD
Director
Division of AIDS Research, National Institutes of Mental Health (NIMH)
5601 Fishers Lane Room 8D20, MSC 9831
Rockville, MD 20852 USA
Phone: 240-627-3874
Fax: 240-627-3467
Email: drausch@mail.nih.gov

Teri Senn, PhD
Program Chief, Psychosocial Co-morbidities of HIV Prevention and Treatment
Division of AIDS Research, NIMH
5601 Fishers Lane Room 9G29
Rockville, MD 20852 USA
Phone: 301-761-7852
Email: teri.senn@nih.gov

Microbicide Trials Network (MTN) Leadership and Operations Center (LOC) – Pitt

Jared Baeten, MD, PhD
Co-Principal Investigator
University of Washington
ICRC, Dept of Global Health
325 Ninth Avenue, Box 359927
Seattle, WA 98104
Phone: 206-520-3808
Fax: 206-520-3831
Email: jbaeten@uw.edu

Katherine Bunge, MD
Protocol Safety Physician
Magee-Womens Hospital of UPMC
300 Halket Street
Pittsburgh, PA 15213 USA
Phone: 412-641-3464
Fax: 412-641-1133
Email: kbunge@mail.magee.edu

Luis Duran, DrPH, MPIA
Project Manager
Microbicide Trials Network
204 Craft Avenue
Pittsburgh, PA 15213 USA
Phone: 412-641-8539
Fax: 412-641-6170
Email: duranl2@mwri.magee.edu

Craig Hendrix, MD
Director, Rectal Microbicide Program and Pharmacology LC Principal Investigator
Johns Hopkins University
600 North Wolfe Street, Harvey 502
Baltimore, MD 21287 USA
Phone: 410-955-9707
Fax: 410-955-9708
Email: cwhendrix@jhmi.edu

Sharon Hillier, PhD
MTN Principal Investigator
Microbicide Trials Network
204 Craft Avenue
Pittsburgh, PA 15213 USA
Phone: 412-641-8933
Fax: 412-641-6170
Email: shillier@mail.magee.edu

Cindy Jacobson, PharmD
Director of Pharmacy Affairs
Microbicide Trials Network
204 Craft Avenue
Pittsburgh, PA 15213 USA
Phone: 412-641-8913
Fax: 412-641-6170
Email: cjacobson@mail.magee.edu

Sharon A. Riddler, MD, MPH
Protocol Physician
UPMC, Keystone Building, Suite 510
3520 Fifth Avenue
Pittsburgh, PA 15213 USA
Phone: 412-383-1741or 412-383-1675
Fax: 412-383-2900
Email: riddler@dom.pitt.edu

Devika Singh MD, MPH
Protocol Safety Physician
19 Randall Drive
Jericho, VT 05465 USA
Phone: 206-920-0975
Email: devika@mtnstopshiv.org

Mei Song, PhD
Protocol Specialist
Microbicide Trials Network
204 Craft Avenue
Pittsburgh, PA 15213 USA
Phone: 412-641-2282
Fax: 412-641-6170
Email: songm4@mail.magee.edu

Jennifer Thomas, MSc
Protocol Development Manager
Microbicide Trials Network
204 Craft Avenue
Pittsburgh, PA 15213 USA
Phone: 412-641-5579
Fax: 412-641-6170
Email: thomasj15@mwri.magee.edu

MTN Laboratory Center (LC)

Edward Livant, BSMT (ASCP), MPH
MTN LC Research Manager
Microbicide Trials Network
204 Craft Avenue
Pittsburgh, PA 15213 USA
Phone: 412-641-3772
Fax: 412-641-5290
Email: livantew@upmc.edu

Lisa Rohan, PhD
LC Representative
University of Pittsburgh Pharmaceutical Sciences
Magee-Women's Research Institute
204 Craft Avenue, Room B509
Pittsburgh, PA 15213 USA
Phone: 412-641-6108
Fax: 412-641-6170
Email: lrohan@mwri.magee.edu

MTN LOC – FHI 360

Kailazarid Gómez-Feliciano, MPM
Senior Clinical Research Manager
FHI 360
359 Blackwell Street, Suite 200
Durham, NC 27701 USA
Phone: 919-544-7040, Ext. 11282
Fax: 919-544-0207
Email: kgomez@fhi360.org

Sherri Johnson, MPH
Senior Clinical Research Manager
FHI 360
1825 Connecticut Avenue, NW
Washington, DC 20009 USA
Phone: 202-884-8000, Ext. 18677
Fax: 202-884-8844
Email: sjohnson@fhi360.org

Jonathan Lucas, MPH
Senior Community Program Manager
FHI 360
359 Blackwell Street, Suite 200
Durham, NC 27701 USA
Phone: 919-544-7040, Ext. 11458
Fax: 919-544-0207
Email: jlucas@fhi360.org

Nicole Macagna, MA
Prevention Research Specialist
FHI 360
359 Blackwell Street, Suite 200
Durham, NC 27701 USA
Phone: 919-544-7040, Ext. 11717
Email: nmacagna@fhi360.org

MTN Statistical Data Management Center (SDMC)

Jennifer Berthiaume, MSW, MPH
Clinical Data Manager
Fred Hutchinson Cancer Research Center (FHCRC) – Statistical Center for HIV/AIDS Research and Prevention (SCHARP)
1100 Fairview Ave. North, J5-000
PO Box 19024
Seattle, WA 98109-1024 USA
Phone: 206-667-1230
Email: jberthia@scharp.org

Elizabeth Brown, ScD
SDMC Principal Investigator
FHCRC
1100 Fairview Avenue North, M2-C200
PO Box 19024
Seattle, WA 98109-1024 USA
Phone: 206-667-1731
Fax: 206-667-4812
Email: erbrown@fredhutch.org

Holly Gundacker, MS
Statistical Research Associate
FHCRC-SCHARP
1100 Fairview Ave North, M2-C200
PO Box 19024
Seattle, WA 98109-1024 USA
Phone: 206-667-6480
Email: hgundack@scharp.org

Karen Patterson, MPH
Program & Portfolio Manager
FHCRC-SCHARP
1100 Fairview Avenue North, E3-129
PO Box 19024
Seattle, WA 98109-1024 USA
Phone: 206-667-7052
Fax: 206-667-4812
Email: karenp@scharp.org

MTN Working Group Representatives

Behavioral Research Working Group (BRWG)

Alex Carballo-Diéguez, PhD
BRWG Representative
HIV Center for Clinical and Behavioral Studies
NYSPI & Columbia University
1051 Riverside Dr., Unit 15
New York, NY 10032 USA
Phone: 646-774-6930
Fax: 646-774-6955
Email: ac72@cumc.columbia.edu

Community Working Group (CWG)

Daniel Gondwe
CWG Representative
Johns Hopkins University Research Project
Chipatala Avenue
P.O. Box 1131
Blantyre, Malawi
Phone: 265-1875-128
Fax: 265-1870-132
Email: dgondwe@jhu.medcol.mw

Matthew Rose
CWG Co-Chair
National Minority AIDS Council
1000 Vermont NW, Suite 200
Washington, DC 20005 USA
Phone: 202-277-2777
Email: mrose@nmac.org

MTN-035

Acceptability, Tolerability, and Adherence of Three Rectal Microbicide Placebo Formulations among HIV Seronegative Cisgender Men, Transgender Men and Transgender Women Who Engage in Receptive Anal Intercourse

INVESTIGATOR SIGNATURE FORM

Version 1.0; June 15, 2018
A Study of the Microbicide Trials Network

Funded by:
Division of AIDS (DAIDS), US National Institute of Allergy and Infectious Diseases
US Eunice Kennedy Shriver National Institute of Child Health and Human Development
US National Institute of Mental Health
US National Institutes of Health (NIH)

IND Sponsor:
A Non-IND Study (DAIDS Protocol ID: 38459)

I, the Investigator of Record, agree to conduct this study in full accordance with the provisions of this protocol and all applicable protocol-related documents. I agree to conduct this study in compliance with United States (US) Health and Human Service regulations (45 CFR 46); standards of the International Council on Harmonisation (ICH) Guideline for Good Clinical Practice (E6); Institutional Review Board/Independent Ethics Committee determinations; all applicable in-country, state, and local laws and regulations; and other applicable requirements (e.g., NIH, DAIDS) and institutional policies.

I agree to maintain MTN study records in accordance with protocol-specified protections of participants' confidentiality and with site IRB/IEC policies and procedures. Study records must be maintained on-site for the entire implementation period of the study and a minimum of at least three years after completion of research as per 45 CFR 46.115 (b). . DAIDS/designee will inform the investigator/institution as to when these documents no longer need to be retained.

I have read and understand the information in this protocol and will ensure that all associates, colleagues, and employees assisting in the conduct of the study are informed about the obligations incurred by their contribution to the study.

____
Name of Investigator of Record

______________________________ ______________________________
Signature of Investigator of Record Date

MTN-035

Acceptability, Tolerability, and Adherence of Three Rectal Microbicide Placebo Formulations among HIV Seronegative Cisgender Men, Transgender Men and Transgender Women Who Engage in Receptive Anal Intercourse

PROTOCOL SUMMARY

**Short Title:**	Rectal Microbicide Acceptability, Tolerability, and Adherence
**Funders:**	Division of AIDS, NIAID, NIMH, NICHD, US NIH
**Protocol Chair**:	José A. Bauermeister, PhD, MPH
**Sample Size:**	MTN-035 will enroll approximately 210 participants
**Study Population:**	HIV-uninfected cisgender men, transgender men (TGM) and transgender women (TGW) aged 18-35 years who engage in receptive anal intercourse (RAI)
**Study sites:**	Sites selected by MTN Executive Committee
**Study Design:**	Multi-site, randomized-sequence, three-period, open label crossover study
**Study Duration:**	Approximately 3.5 months of follow-up with a projected accrual period of 9-12 months
**Study Products:**	Placebo rectal insert<br>Placebo rectal douche<br>Placebo rectal suppository
**Study Regimen:**	Participants will be randomized (1:1:1:1:1:1) to study product sequences A-F (see Table 1 below). At the start of each 4-week product use period, they will receive either rectal inserts, rectal douches, or rectal suppositories and be instructed to use their assigned study product prior to each RAI encounter during that period. Participants who do not have RAI in a given week will be asked to use the product without sex. There will be a 1-week washout period between each of the three product use periods.

Table 1: MTN-035 Study Product Regimen

Sequence	N	Period 1 (4 weeks)	Washout period (~1 week)	Period 2 (4 weeks)	Washout period (~1 week)	Period 3 (4 weeks)
A	35	Rectal insert	--	Rectal douche	--	Rectal suppository
B	35	Rectal douche	--	Rectal suppository	--	Rectal insert
C	35	Rectal suppository	--	Rectal insert	--	Rectal douche
D	35	Rectal insert	--	Rectal suppository	--	Rectal douche
E	35	Rectal douche	--	Rectal insert	--	Rectal suppository
F	35	Rectal suppository	--	Rectal douche	--	Rectal insert

Figure 1: MTN-035 Study Visit Schedule

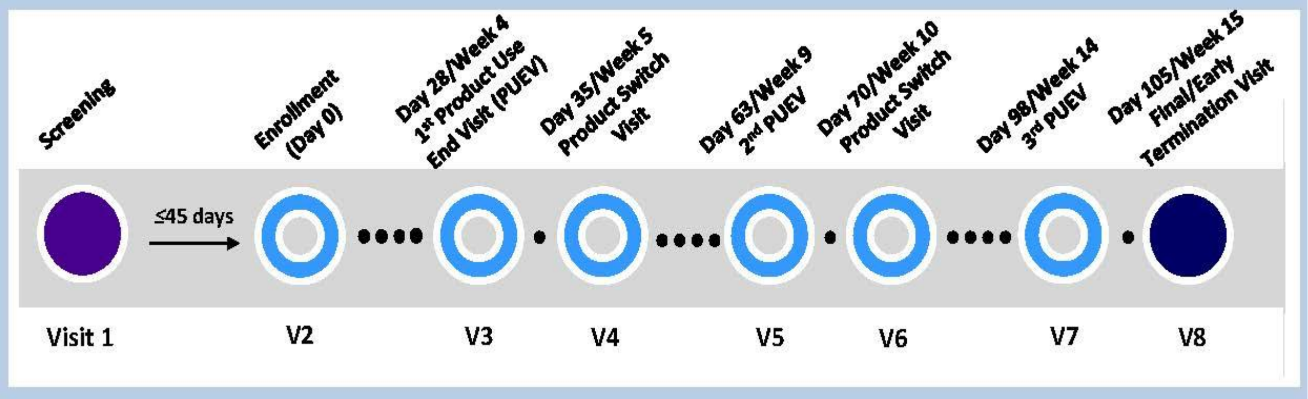

Primary Objectives:

Acceptability and Tolerability

- To evaluate the acceptability and tolerability of each study product when applied rectally and used prior to RAI
  - Placebo rectal insert
  - Placebo rectal douche
  - Placebo rectal suppository

Adherence

- To evaluate adherence to each study product prior to RAI over a 4-week-long period
  - Placebo rectal insert
  - Placebo rectal douche
  - Placebo rectal suppository

Safety

- To evaluate the safety of each study product when applied rectally and used prior to RAI
  - Placebo rectal insert
  - Placebo rectal douche
  - Placebo rectal suppository

Exploratory Objective:

Relative Acceptability and Tolerability

- To evaluate the relative acceptability and tolerability between study products when applied rectally and used prior to RAI
  - Placebo rectal insert
  - Placebo rectal douche
  - Placebo rectal suppository

Primary Endpoints:

Acceptability and Tolerability

- For each study product, participant self-report of likelihood of product use if shown to be effective

Adherence

- Per participant report, percentage of occasions when each study product was used as instructed (per protocol)

Safety

- Grade 2 or higher related adverse events (AEs) as defined by the Division of AIDS Table for Grading the Severity of Adult and Pediatric Adverse Events, Corrected Version 2.1, July 2017, and/or Addenda 1, 2 and 3 (Female Genital [Dated November 2007], Male Genital [Dated November 2007] and Rectal [Clarification Dated May 2012] Grading Tables for Use in Microbicide Studies)

Exploratory Endpoint:

Relative Acceptability and Tolerability

- Conjoint analysis of participant acceptability and tolerability between the three study products

1 KEY ROLES

1.1 Protocol Identification

Protocol Title:	Acceptability, Tolerability, and Adherence of Three Rectal Microbicide Placebo Formulations among HIV Seronegative

Cisgender Men, Transgender Men and Transgender Women Who Engage in Receptive Anal Intercourse

Protocol Number: MTN-035

Short Title: Rectal Microbicide Acceptability, Tolerability, and Adherence

Date: June 15, 2018

1.2 Funding Agencies and Sponsor Identification

Funding Agencies: US Division of AIDS (DAIDS)/National Institute of Allergy and Infectious Diseases (NIAID)
National Institutes of Health (NIH)
5601 Fishers Lane
Rockville, MD 20852 USA

US National Institute of Mental Health (NIMH)
6001 Executive Boulevard
Rockville, MD 20852 USA

US *Eunice Kennedy Shriver* National Institute of Child Health and Human Development (NICHD)
Maternal and Pediatric Infectious Diseases (MPID) Branch
6100 Executive Boulevard
Rockville, MD 20852 USA

Pharmaceutical Company Collaborator: CONRAD
1911 North Fort Myer Drive, Suite 900
Arlington, VA 22209 USA

1.3 Medical Officer

Medical Officer: Jeanna Piper, MD
DAIDS/NIAID
5601 Fishers Lane, Room 8B68, MSC 9831
Rockville, MD 20852

1.4 Clinical Laboratories

Network: MTN Laboratory Center (LC)
204 Craft Ave.
Pittsburgh, PA 15213 USA

1.5 Data Centers

Data Center: MTN Statistical Data and Management Center (SDMC)
Statistical Center for HIV/AIDS Research & Prevention
(SCHARP)/Fred Hutchinson Cancer Research Center
(FHCRC)
1100 Fairview Avenue N., LE-400
PO Box 19024
Seattle, WA 98109-1024 USA

Qualitative Data Center: University of Pennsylvania
418 Curie Blvd.
Philadelphia, PA 19104

1.6 Study Implementation

Study Operations: MTN LOC - FHI 360
359 Blackwell Street, Suite 200
Durham, NC 27701 USA

2 INTRODUCTION

2.1 Background of Rectal Microbicide Research and Study Rationale

Microbicides are products that are designed to be applied to the vaginal or rectal mucosa with the intent of preventing the acquisition of sexually transmitted infections (STIs) including the human immunodeficiency virus (HIV).[1] The original impetus for microbicide development was to provide women with options for HIV prevention in settings where their partners were unwilling to use condoms for penile-vaginal intercourse. However, there is recognition that rectal microbicides are needed for men and women who practice receptive anal intercourse (RAI).

RAI is associated with the highest probability for sexual acquisition of HIV infection. Unprotected RAI is the sexual behavior with the highest per act risk of HIV acquisition, conferring approximately 10 to 20 times more risk than unprotected receptive vaginal intercourse (RVI).[2,3] Globally, transgender women (TGW) and men who have sex with men (MSM) are 19 times more likely to be living with HIV compared with the general population.[4,5]

Despite scaled up prevention efforts and expanding access to antiretroviral (ARV) treatment in the last few decades, HIV rates remain disproportionately high among MSM throughout the world, with prevalence ranging from a low of 3.0% in the Middle East and North Africa to a high of 25.4% in the Caribbean.[6] In low and middle income countries in Africa, Asia and Latin America, MSM continue to experience high rates of HIV infection.[6] [7]In European Union/European Economic Areas, HIV diagnoses among MSM declined in specific countries, such as the United Kingdom, Belgium, Italy, the Netherlands and Spain, but HIV continues to increase among MSM in other EU/EEA countries, with substantial increases in Bulgaria, Croatia, Cyprus, Czech Republic, Hungary, Ireland, Lithuania, and Malta in recent years.[8] In the US, according to CDC, estimated annual HIV infections remained stable among all gay and bisexual men during 2010 to 2014, but with increase among MSM aged 25 to 34 and Hispanic/Latino MSM.[9] As modeled by Stall et al (2009), even incidence rates as low as 2-3%, like those observed among MSM in high-income countries between 1995 and 2005, are likely to yield prevalence rates >40% within 25 years for a cohort of 15-year old gay males in the US.[10]

For the most vulnerable MSM populations, the likelihood of HIV infection is even higher. For example, black MSM in the US are 15 times more likely to be HIV positive than the general population and 8.5 times more likely than other African Americans.[11] In African countries that criminalize homosexual activity, black MSM are roughly 72 times more likely to become infected with HIV than the general population in the US, and over 100 times more likely than the general population in the UK.[11] In South Africa, rates among black MSM are much higher than the general population, with 43.6% HIV prevalence among gay participants in one study conducted in Johannesburg and Durban, and 49% HIV prevalence among gay participants aged 20-24 years old in another study.[12] In

Kenya, the only African country with HIV incidence data, annual HIV incidence was reported at >20% in Mombasa.[6]

Another segment of the population facing disproportionately high levels of HIV are transgender women (TGW) and transgender men (TGM). One meta-analysis estimated that HIV prevalence for TGW was 19.1% worldwide, with 21.6% for TGW in high-income countries (including 22% of 2,705 TGW sampled in the US) and 17.7% in middle- and lower-income countries.[13] The same study found that the risk of HIV infection was 50 and 46.3 times higher, respectively, among TGW than the general population of reproductive-aged adults in middle- and lower-income countries and in higher income countries. Of the 2351 transgender people diagnosed with HIV between 2009 and 2014 in the US, 84% were TGW, 15% were TGM, and <1% identified with another gender.[14] Similar to MSM, a meta-analysis found even higher HIV infection rates (30.8% to 56.3%) among black TGW than the general TGW population.[15]

Transgender sex workers experience even higher HIV infection rates. A systematic review found that overall crude HIV prevalence was 27.3% among TGW engaging in sex work, compared to 14.7% of those not engaging in sex work.[16] This same meta-analysis indicated that transgender sex workers experienced significantly higher risk for HIV infection in comparison to all other groups (RR=1.46), particularly in comparison to female sex workers (RR=4.02). However, little data about HIV prevalence is available for TGM or other transgender populations.

The World Health Organization (WHO) developed and encourages the use of a combination approach to combat the HIV epidemic in key populations, including the gay and transgender communities. It includes: comprehensive condom and lubricant programs, harm reduction for injection drug users, behavioral interventions, ARV-related prevention and voluntary medical male circumcision (VMMC), HIV testing and counselling, HIV treatment and care including prevention of mother-to-child transmission (PMTCT), prevention of coinfections like tuberculosis and hepatitis, prevention and management of mental health problems, and other general care related to sexual and reproductive health and nutrition.[17]

However, prevention services are often grossly inadequate, even in high-income countries. For example, a 2010 cross-European Internet survey estimated that only 25–50% of MSM were tested for HIV infection the previous year, compared with 60% in Australia and 77% in the US.[18] In low and middle income countries, prevention needs among MSM have also been neglected due to limited resources and inadequate service quality, especially in countries where male-to-male sexual activity is stigmatized or criminalized.[19,20] Based on a UN General Assembly Special Session (UNGASS) report, it is estimated that only a third of MSM in the 23 low to middle income countries surveyed were reached by HIV prevention programs.[21] Furthermore, research suggests that black MSM are less likely to have received free condoms and lubricants than other MSM[22], less likely to be tested for HIV than white MSM[23], and reported fewer service locations in their neighborhood.[24]

Condoms are highly cost-effective tools for prevention of HIV, STIs and unintended pregnancies. But, condoms are not readily available in some countries, especially in sub-Saharan Africa. The Joint United Nations Programme on HIV/AIDS (UNAIDS) estimated the need for male condoms was 6 billion in 47 sub-Saharan countries in 2015, yet only an estimated 2.7 billion condoms were distributed in those countries that year. This suggests over half the condom need in the region is not being met, with South Africa as the only country surpassing the condom needs of its people. And, when condoms are accessible, they are not consistently used. UNAIDS also estimated that only three of 104 countries with available data reported >90% condom use at last sexual intercourse among MSM.[25]

There is little data on condom use among transgender people worldwide, but limited evidence shows that condom use in this population is low. A study in Cambodia found that consistent condom use by transgender people was higher (40%) with female partners than with male partners (20–40%), with the lowest rates of use with male partners when buying sex.[26] Another study in Thailand found that 46.7% of MSM and 52.3% of transgender people used condoms in the past three months.[27]

Analysis of CDC's National HIV Behavioral Surveillance (NHBS) data suggests that condom use among US MSM declined 20% from 2005 to 2011.[28] Increases in condomless sex have also been reported in other developed countries.[29-31] Research suggests that the increase in condomless sex has offset the reduction in HIV incidence due to earlier HIV diagnosis and ART among MSM.[32,33] In a follow-up analysis of the NHBS data with new data from 2014, Paz-Bailey and colleagues investigated if the increases in condomless sex were associated with use of other prevention strategies, and found that decreased condom use was not explained by serosorting, seropositioning, PrEP use or HIV treatment.[34] However, another study found that condomless anal intercourse (AI) among its MSM and TGW participants decreased during follow-up regardless of whether they received PrEP or not.[35]

There will always be people who either prefer sex without a condom (e.g., due to increased sexual pleasure, erectile dysfunction, intimacy, or spontaneity), or who do not have the necessary autonomy to negotiate condom use with their partners (e.g., sex workers, adolescents and young adults, alcohol and drug users). This is evident from various analyses of national survey data suggesting low condom use rates among both men and women who engaged in RAI (44% among males and 11% among females for most recent RAI encounter, and 26% among males and 13% among females for past 10 RAI encounters).[36-38] This data highlights the unrealistic expectation for consistent condom use. HIV prevention alternatives are needed to accommodate all persons at risk of HIV infection, particularly persons who engage in disproportionately risky activities like RAI.

Currently, there is one biomedical alternative to condoms that is approved for HIV prevention, the Truvada oral tablet. Truvada for oral pre-exposure prophylaxis (PrEP), when used consistently and as indicated, significantly reduces the risk of HIV acquisition and is generally safe.[39] Also, treating the infected partner in an HIV-serodiscordant couple

with combination antiretroviral therapy (ART) including Truvada can reduce HIV transmission by up to 96%.[40] Furthermore, studies have consistently found no association between PrEP use and changes in risky sexual behavior[41] or hormonal contraception effectiveness.[42,43]

In the iPrEx study, self-reported oral PrEP adherence among MSM and TGW was high (≥89% at week 4), but drug concentrations in participants suggested that actual adherence was probably lower.[44] Another open-label study found that PrEP uptake and adherence among MSM and TGW was high when participants had access to PrEP free of charge from experienced health providers.[35] In contrast, however, population surveys of MSM suggest that overall PrEP use levels are still low[45,46], with reported barriers such as low PrEP awareness[46] and lack of knowledge and prescribing experience among health providers.[47]

Although oral PrEP holds great promise for HIV prevention, its scale and coverage remain limited outside the US. UNAIDS estimates that 60,000 people were taking oral PrEP in 2016, with most of them in the US, though this estimate does not include those who access PrEP through less regulated channels like the Internet. In Africa, UNAIDS identified several barriers to scaling up PrEP use, including cost, limited awareness, limited regulatory approval (currently approved in only seven countries worldwide), and limited availability of both PrEP and the ancillary clinical services needed to support its successful implementation.[25]

While generally effective for use as oral PrEP, Truvada tablets require a daily dosage schedule to maintain protective levels over time.[39,48] Also, while Truvada is generally safe for use as oral PrEP, systemic delivery of a drug increases the risk and severity of its possible side effects relative to more localized drug delivery alternatives like microbicides. Lastly, behaviorally and conceptually linking the taking of pills (a daily prevention strategy) with sexual activity (an episodic occurrence) could be difficult for those who would find a pericoital prevention strategy more feasible and/or preferable. Therefore, even if readily available to everyone, such product characteristics could lead to acceptability and adherence challenges for many, and do not address other STIs that can still be passed via unprotected RAI.

It is important to develop a range of HIV prevention products that can serve as viable alternatives and/or complements to consistent condom use and oral PrEP, including formulations able to deliver multiple drugs (e.g., anti-HIV-1 and anti-HSV-2) in combination. There are many other drugs being developed for HIV prevention in various formulations, including intravaginal rings (IVR), topical gels, film inserts, and injectables.[1,49,50] However, as past PrEP and microbicide studies have shown, drug efficacy translates to drug effectiveness only when people use the drug as intended.[51-53]

MSM have expressed a preference for topical gel microbicides over other rectal microbicide formulations because similarities to personal lubricants would mean that a gel microbicide could potentially be readily incorporated into their usual sexual practices.[33,54,55] Unfortunately, microbicide gel formulations tested thus far have required

the use of an applicator which could present acceptability and adherence challenges for long-term use.[56] Although a lubricant rectal microbicide product would seem an ideal drug delivery mechanism from an acceptability and adherence standpoint, it is unclear if topical gels could be developed to deliver protective levels of HIV prevention drugs when used without an applicator.

It is crucial for drugs to be designed so that they can be delivered via mechanisms that not only deliver enough drug to block HIV transmission, but are also a good behavioral fit with the drug's intended end-users. MTN-035 will evaluate the acceptability of three potential rectal microbicide formulations – an insert, a douche, and a suppository – that could be used prior to RAI and could potentially deliver enough anti-HIV drug locally to prevent HIV infection during RAI. It is believed these drug delivery mechanisms would be congruent with cleansing practices and behaviors regarded as normal preparations prior to engaging in RAI or participating in activities where the probability of engaging in RAI is high.[57,58]

2.2 Description of Study Products

2.2.1 Placebo rectal insert

The placebo rectal insert is formulated as a white to off-white solid dosage form. The insert is small (about 1.5 cm [2/3 of an inch] long and about 0.7 cm [1/3 of an inch] wide) and bullet-shaped. The rectal insert evaluated in this study will be a placebo product intended for use without an applicator.

Safety of placebo rectal insert

Rapidly disintegrating topical inserts offer many advantages as on-demand drug dosage forms: inexpensive, portable, easy to apply, discreet, user-initiated, and suitable for both vaginal and rectal application. CONRAD developed four placebo insert prototypes with varied preparations, formulations, weights, and shapes, including the placebo insert being evaluated in this study.[59]

Toxicity studies were conducted with rabbits and with the EpiVaginal VEC-100-FT human tissue model to evaluate the potential for local irritation of various active and placebo inserts, including the placebo insert being evaluated in this study. Following once-daily rectal application in rabbits for 14 days, there were no definitive article-related effects observed for any of the test parameters: bodyweight, clinical observations, macroscopic or microscopic tissue pathology. All observed effects were deemed to be procedural in origin, not due to the test articles. The average rectal irritation index for the placebo insert was <1, indicating no local irritation. Following a 24-hour exposure to the placebo insert in the EpiVaginal human tissue model, histopathology results showed there were no significant changes to tissue morphology, with only a thinning of the epithelium while the basal and parabasal cell layers remained intact. The placebo insert did not induce any noticeable effects on tissue viability (110% by MTT assay), barrier integrity (transepithelial electrical resistance [TEER] = 95.3%), or tissue structure.[59]

There are no human data for rectal use of the CONRAD placebo insert. The CONRAD 134 study tested the safety, stability, and disintegration profile of four inserts when administered vaginally by healthy women aged 18-50. All four placebo inserts were found to be safe during 1 or 2 uses, with only one mild AE reported which was assessed as unrelated to study product. The four placebo inserts also disintegrated faster, had less residue leakage, and were more acceptable than the first-generation placebo inserts previously used in CONRAD 117, a Phase 1 safety and PK study of TFV, FTC, and TFV/FTC vaginal inserts. Of the four products tested in CONRAD 134, Placebo Insert C was selected for evaluation in this study.[59] The selection of Placebo Insert C was based on the following criteria as evaluated in the CONRAD-134 study: disintegration, acceptability, physiochemical stability, and preliminary cost-of-goods analysis. As compared to Placebo Inserts A, B, and D, Placebo Insert C had favorable results for all study benchmarks: it achieved improved disintegration and acceptability rates as compared to first generation inserts, showed no changes in appearance or hardness at accelerated storage conditions (40˚C/75%RH), and achieved feasibility of meeting price targets. (Personal communication with CONRAD, March 23, 2018)

Initial disintegration of the placebo insert in CONRAD 134 occurred quickly, with median values of 7.5 minutes for Placebo Insert C. At 60 minutes, 67% (8/12) of Placebo Insert C users had less than half the insert visible on exam. At 45 minutes, 25% (3/12) had less than half visible on exam. It is expected the insert will disintegrate faster during rectal use with the addition of intercourse, somewhere between the results seen in CONRAD 134 and the results from in-vitro dissolution testing. In in-vitro dissolution testing at 37°C, approximately 42% of the insert dissolved by 5 minutes, approximately 94% of the insert dissolved by 15 minutes, and approximately 98% of the insert had dissolved by 60 minutes.

2.2.2 Placebo rectal douche

The rectal douche evaluated in this study will consist of approximately 120 mL of water administered rectally via a disposable, commercially available 125 mL enema bottle.

Safety of rectal douches

Rectal enemas have long been used clinically as bowel preparations before procedures and therapeutically for constipation and intussusception. Investigators at the University of Washington studied the rectal mucosal safety of various enema preparations, including Fleet’s Phospho-Soda, Bisacodyl (Dulcolax), and a 500 mL 0.9% (iso-osmolar, normal) saline enema.[60] The study evaluated proctoscopic appearance, light microscopic appearance, and scanning and transmission electron microscopy of rectal biopsies obtained via sigmoidoscopy 5 to 10 cm proximal to the anus, within 10 minutes after the various enema agents were administered and expelled. Proctoscopically, there were no abnormal findings after the saline enema, but some vascular disruption was grossly observed after Fleet's or Bisacodyl enema in all subjects. On light microscopy, only 3 of 21 (14%) subjects who were given the saline enema were seen to have abnormalities at

the epithelial surface, but no abnormalities were observed in the epithelial crypt or lamina propria, whereas much greater microscopic epithelial disruption was observed after Fleet's enema (81%, or 17 of 21 biopsies) and Bisacodyl enema (84%, or 21 of 25 biopsies). Of the 3 of 21 (14%) biopsies after saline enema that were observed to have an absent superficial epithelial layer, the appearance was consistent with an abrasion possibly caused by either sigmoidoscope or enema tip. When the biopsies were evaluated by scanning electron microscopy, all 5 biopsies from subjects who had received the saline enema had a normal appearance, whereas the biopsies from individuals who had received Fleet's enema had absent goblet cells, abnormal microvilli, or disrupted surface in 6 of 7 (86%) cases. Overall, the proctoscopic and scanning electron microscopic appearance of rectal mucosa after saline enema was no different than that observed in control participants.

Integrated Preclinical/Clinical Program (IPCP) Microbicide Development Program's (MDP) Project 5/Aim 1[61] studied the safety and acceptability of three enema microbicide vehicles and was performed to elucidate the preferred osmolarity of a rectal tenofovir enema. In this study, investigators assessed the distribution, safety, and acceptability of three enema formulations: hyper-osmolar (Fleet), hypo-osmolar (distilled water), and iso-osmolar (Normosol-R), using a crossover design. For Project 5/Aim 1, SPECT/CT imaging was performed after administration of radiolabeled enema product. This imaging showed that the iso-osmolar enema had greater colonic distribution (up to the splenic flexure) and greater luminal and colonic tissue concentrations of radiolabeled product compared to the other 2 enema formulations ($p<0.01$). Colonic biopsies were also performed, and demonstrated that colonic epithelial sloughing was induced only by the hyper-osmolar enema ($p<0.05$) and not by the other two enema products. Lastly, in permeability studies of the radiolabeled diethylenetriaminepentaacetic acid (DTPA) 9 used primarily for luminal imaging, the hypo-osmolar enema resulted in the highest plasma area under the concentration-time curve (AUC) and peak concentration of radiolabeled enema out of the three enema types.

The rectal douche evaluated in this study, an enema filled with bottled or tap water, is expected to be as safe as the distilled water and saline enemas described above.[61,62]

2.2.3 Placebo rectal suppository

The rectal suppository evaluated in this study will be a 2 g, 3-3.8 cm (1.2-1.5 inches) long, fat-based, fast-release suppository intended for use without an applicator.

Safety of placebo rectal suppositories

The suppository will consist of Witepsol® H5 (IOI Oleocehmical), a commonly used suppository base. Witepsol® H5 is a hard fat suppository base majorly composed of triglycerides with 15% diglyceride and not more than 1% monoglyceride content. Witepsol® suppository bases are commonly utilized in several marketed products by various manufacturers. The table below lists the Witepsol®-based suppository products currently on the market[63]:

Table 2: Witepsol® H5-based suppository products currently on the market

Brand Name	Active ingredients	Manufacturer
Alvedon	Paracetamol	GSK (producer Kemwell)
Anodesyn	Lidocaine + Allantoin	Thornton & Ross = Stada
Asacol	Mesalazine	Warner Chilcott Actavis
Bisacodyl	Bisacodyl	Petrus Australia
Bismuth	Bismuth Subgallate	Martindale
Boots Haemorrhoid	Allantoin, Lidocaine	Boots
Doloproct/Ultraproct	Fluocortolon + Lidocaine	Bayer
Dulcolax	Bisacodyl	Boehringer Ingelheim
Canasa	Mesalamine	Allergan/Aptalis
Eucalyptine	Codein + Eucalyptol	Medgenix
Febricet	Paracetamol	Hemopharm
Flagyl	Metronidazole	Zentiva
Germoloids	Zinc Oxide, Lidocaine	Bayer
Imigran	Sumatriptan	GSK
Jelliproct	Fluocinonid + Lidocaine	Teofarma
Kortos	Hydrocortisone	Embil
Mesasal	Mesalazine	Sanofi
Morphine Sulphate	Morphine	Aurum UK
Nurofen Junior	Ibuprofen	Reckitt Benckiser (Famar)
Procto-Glyvenol	Tribenoside, Lidocaine	Recordati
Salazopyrin	Sulfasalazine	Pfizer
Tramadol Stada	Tramadol	Stada
Uniroid HC	Hydrocortisone + Cinchocaine	Chemidex (Famar)
Voltaren	Diclofenac	Novartis
Vomacur	Dimenhydrinate	Hexal
Zofran	Ondansetron	GSK

2.3 Acceptability of non-gel rectal microbicide formulations

2.3.1 Rectal inserts/suppositories

There is little data available on the use of rectal inserts or suppositories specifically by cisgender men, TGM and TGW. One study assessing rectal health and behaviors among 896 HIV positive and negative men and women in Los Angeles and Baltimore found that 38% (101/263) of male and 40% (85/211) of female participants reported ever using a rectal suppository.[64]

Studies have been conducted with MSM in various countries to assess potential users' preference and acceptability for various rectal microbicide delivery methods, including gel, cream, suppository and enema. One qualitative study in India found that gel was the preferred formulation among focus group participants, consistent with participants' use of water-based lubricants.[65] One survey study in Thailand (n = 408) found that young MSM and TGW preferred the gel formulation over suppository (odds ratio = 1.4, p <0.01).[66]

Similarly, one randomized trial in the US found higher acceptability for a gel microbicide formulation over rectal suppository (75% vs. 25%, p<0.001, n=77).[55] Lastly, a survey study (n = 350) assessing rectal microbicide acceptability among MSM in Tianjin, China found the highest acceptability for a formulation that "makes your rectum wetter than normal during sex", while a tablet or suppository formulation was viewed as more acceptable than a "jelly-like" formulation.[67]

Though suppositories are viewed as less desirable than other formulations, they are still viewed as a viable rectal microbicide delivery method.[56] Suppositories are very effective in delivering medication to the rectum. Brown et al (1997) compared suppositories against enemas and foams in their delivery of mesalazine, and only with suppositories was the spread of mesalazine confined to the rectum during 4 hours of gamma scintigraphy, while enemas and foams both spread the drug outside the rectum to the colon.[68] Also, there have been no studies evaluating suppositories specifically formulated to deliver rectal microbicides. For example, the suppository used in the randomized US trial mentioned earlier[55] was much larger (8 g, 6.4 cm [2.5 inches] long) than the average adult suppository in the market (2 g, 3 cm [1.2 inches] long) or the suppository planned for this study (2 g, 3-3.8 cm [1.2-1.5 inches] long). It is expected that suppositories which are smaller, dissolve more readily and/or are easier to insert may result in different acceptability ratings.

2.3.2 Rectal douches

Rectal douching is a common cleansing practice for cisgender men, TGM and TGW who engage in RAI (see Section 2.4 for a review of AI-related douching practices). Most MSM who douche rectally use water or other homemade solutions, and most of those who use commercial products tend to use saline-based solutions.[57,69,70] In a survey conducted in the US, the type of douching solution used varied between racial/ethnic groups, with water being most commonly reported by White participants before (73.8%) and after (64.5%) AI, whereas water added with salt, soap, or some other antibacterial product was most commonly reported by Hispanic participants before and after AI, and commercially-prepared saline solution was most commonly reported by Black participants.[70] Likewise, a wide range of devices were used for rectal douching, including showerheads, plastic soda bottles, hair dye bottles, commercial enema kits and syringes.[71,72]

A few studies have explored the acceptability of rectal douches among MSM in different countries. In one US study, 63% of the male couples surveyed (n = 333 couples) indicated a willingness to use rectal microbicides in the form of a douche before AI if proven effective for HIV prevention, and this willingness did not differ significantly by HIV status.[73] A French study also found that 58.5% of MSM who reported recent rectal douche use were likely or very likely to use a hypothetical rectal microbicide douche for HIV prevention, compared to 49.2% of MSM who did not report recent rectal douche use.[74] Similar support for rectal microbicide douche formulations has been reported in developing countries. Galea et al (2014) conducted a focus group study with MSM in Peru and Ecuador (n = 140), where participants voiced support for a douche-based rectal microbicide because it could serve as both a cleansing and HIV prevention tool. They

also felt that a douche would provide greater protection than a lubricant as it could go deeper into the body, and proposed potential drawbacks to a rectal microbicide douche formulation including possible discomfort from an internal cleansing, lack of time to use the douche prior to sex, and the inconvenience of carrying the douche around.[69]

2.4 Practices commonly associated with RAI

One common practice associated with AI is the use of lubricant during sex. An international Internet-based survey found that over 59% of respondents (n = 6124) reported consistent lubricant use[75], while a US-based study found that 94% of MSM surveyed (n = 307) used lubricant when they had anal sex and 74% used lubricant at least in 80% of anal sex occasions.[76] In another US-based study, among young MSM who reported lubricant use (n = 91), 88% of participants used water-based products, 33% used silicone-based products and 14% used oil-based products.[77] Water-based lubricants are compatible with condoms and may provide additional prevention against STIs and HIV because they reduce condom breakage and rectal trauma.[78] However, men without access to water-based lubricants may sometimes use saliva, body cream or petroleum jelly, which increase the risk of condom failure and viral transmission.[79] Using lubricant without condoms has also been linked to an increased risk of STIs.[80]

Rectal douching is another common practice for cisgender men, TGM and TGW who engage in RAI. One recent international study found that 62% of participants (n = 1725) who had ever engaged in AI douched for cleansing purposes, and 83% of those (n = 1070) douched always or most of the time before AI.[57] Another study found that 52% of US MSM surveyed (n = 4992) had douched at least once in their lifetime, with 35% of respondents having douched in the last three months.[70] For those who had douched at least once in their lifetime (n = 2597), that same study found no significant difference across racial/ethnic groups in prevalence of douching before RAI, but among those who reported douching after RAI (n = 711), there was a 2-3-fold higher likelihood of non-White MSM douching after RAI than White MSM (43%-56% vs. 19%, p<0.001). In their survey of Peruvian MSM and TGW, Galea and colleagues found that 18% of participants (n = 415) reported rectal douching in the previous 6 months.[71] In France, 54.3% of MSM surveyed (n = 580) reported using a rectal douche in the past 3 months[74], while in Brazil, 53.4% of gay and transgender persons interviewed who engaged in AI in the previous 3 months (n = 369) used a rectal douche.[72]

One study of MSM living in New York City (n = 105) found that the average age of onset for douching before AI was 25 years old, and that higher proportions of HIV-positive vs. HIV-negative MSM douched before AI (96% [26/27] vs. 53% [41/78]) and after AI (44% [12/27] vs. 27% [21/78]).[58] Multiple studies have identified significant associations between engaging in rectal douching and having HIV and/or an STI[57,70,74], using substances during sex[57], participating in group sex[74], and being “versatile”, i.e., engaging in both insertive and receptive AI.[71]

Reasons for douching before RAI include: a desire to maintain personal hygiene, a desire to adhere to a sexual partner’s request, a desire to prepare or get excited for RAI, and a

belief that douching enhances pleasure during RAI.[57,58,69,70,81] Reasons for douching after sex include: a belief that the practice may help protect them against STIs, a belief that douching following RAI enhances pleasure, a desire to maintain personal hygiene, a desire to transition from sex, and a desire to adhere to a sexual partner's request.[58,69,70,81] MSM typically report douching about one hour prior to an expected RAI encounter and about thirty minutes following RAI.[70,81]

No data exists regarding the use of suppositories or inserts prior to RAI. MTN-035 will be the first study to assess those two formulations in this context, and study staff will document RAI practices in fine detail as part of this assessment. One topic of exploration will be what participants do before and after administering the suppositories or inserts, including whether participants douche prior to administering these two formulations. These findings will allow researchers to develop evidence-based product use recommendations and restrictions in the event the development of these formulations moves into safety trials.

2.5 Study Hypotheses and Rationale for Study Design

2.5.1 Study Primary Hypotheses

It is hypothesized that the placebo rectal insert, the rectal douche, and the rectal suppository will be safe, acceptable and tolerable to participants when administered rectally and used prior to engaging in RAI. It is also hypothesized that participants will use the placebo rectal insert, the rectal douche, and the rectal suppository as instructed during the 4 weeks they are assigned to use each product.

2.5.2 Rationale for Study Design

Rectal microbicides are needed for individuals at risk of acquiring HIV infection through RAI, particularly young gay men and TGW. It is important to expand the rectal microbicide pipeline through the addition of products from different classes that are delivered through various mechanisms, including formulations able to deliver multiple drugs in combination. Many people who engage in RAI also engage in a variety of cleansing and preparatory practices around RAI encounters. Furthermore, intermittent pericoital dosing of rectal microbicides associated with sexual activity may be a more feasible long-term HIV prevention strategy for some people than daily dosing with oral tablets. Therefore, the use of on-demand rectal microbicides like the insert, douche and suppository have the potential advantage of both familiarity and context. Data are needed on the acceptability, tolerability, and adherence to these three formulations and to this dosing schedule among cisgender men, TGM and TGW.

MTN-035 participants will be randomized to one of six sequences of a rectal insert, a rectal suppository, and a rectal douche as per Table 1. Each study product will be administered prior to RAI for four weeks, with a one-week washout period between the three product use periods. This crossover design allows for the comparison of acceptability, tolerability and adherence data within individuals who have been exposed

to each of the three study products when applied rectally and used prior to RAI.

3 OBJECTIVES

3.1 Primary Objectives

Acceptability and Tolerability

- To evaluate the acceptability and tolerability of each study product when applied rectally and used prior to RAI
  - Placebo rectal insert
  - Placebo rectal douche
  - Placebo rectal suppository

Adherence

- To evaluate adherence to each study product prior to RAI over a 4-week-long period
  - Placebo rectal insert
  - Placebo rectal douche
  - Placebo rectal suppository

Safety

- To evaluate the safety of each study product when applied rectally and used prior to RAI
  - Placebo rectal insert
  - Placebo rectal douche
  - Placebo rectal suppository

3.2 Exploratory Objective

Relative Acceptability and Tolerability

- To evaluate the relative acceptability and tolerability between study products when applied rectally and used prior to RAI
  - Placebo rectal insert
  - Placebo rectal douche
  - Placebo rectal suppository

4 STUDY DESIGN

4.1 Identification of Study Design

MTN-035 is a multi-site, randomized-sequence, three-period, open label crossover study.

4.2 Summary of Major Endpoints

Primary Endpoints:

Acceptability and Tolerability

- For each study product, participant self-report of likelihood of product use if shown to be effective

Adherence

- Per participant report, percentage of occasions when each study product was used as instructed (per protocol)

Safety

- Grade 2 or higher related adverse events (AEs) as defined by the Division of AIDS Table for Grading the Severity of Adult and Pediatric Adverse Events, Corrected Version 2.1, July 2017, and/or Addenda 1, 2 and 3 (Female Genital [Dated November 2007], Male Genital [Dated November 2007] and Rectal [Clarification Dated May 2012] Grading Tables for Use in Microbicide Studies)

Exploratory Endpoint:

Relative Acceptability and Tolerability

- Conjoint analysis of participant acceptability and tolerability between the three study products

4.3 Description of Study Population

The study population will consist of HIV-uninfected cisgender men, TGM and TGW aged 18-35 who engage in RAI and meet the criteria outlined in Sections 5.2 and 5.3.

4.4 Time to Complete Accrual

The time to complete accrual is anticipated to be approximately 9-12 months.

4.5 Study Groups

MTN-035 will enroll approximately 210 participants randomized (1:1:1:1:1:1) to one of six sequences of rectal microbicide product application.

4.6 Expected Duration of Participation

Each participant will be in the study for approximately three and a half months. The total duration of the study will be approximately 13-16 months.

4.7 Sites

Sites selected by the MTN Executive Committee.

5 STUDY POPULATION

5.1 Selection of the Study Population

The inclusion and exclusion criteria in Sections 5.2 and 5.3 will be used to ensure the appropriate selection of study participants.

5.1.1 Recruitment

Participants will be recruited from a variety of sources, including outpatient clinics, universities, community-based locations, online websites and social networking applications. In addition, participants may be referred to the study from other local research projects and other health and social service providers. Recruitment materials will be approved by site Institutional Review Boards/Independent Ethics Committees (IRBs/IECs) prior to use. Advice regarding these materials will be sought from site community representatives before they are submitted to the site IRB/IEC for review.

5.1.2 Retention

Once a participant is enrolled and randomized in MTN-035, the study site will make every effort to retain the participant in follow-up to minimize possible bias associated with loss-to-follow-up. A retention rate of 95% will be targeted. The site will be responsible for developing and implementing local Standard Operating Procedures (SOPs) to target and ensure high rates of retention.

5.2 Inclusion Criteria

Individuals who meet the following criteria are eligible for study inclusion:

1. Men (cis or transgender) and TGW who are 18-35 years old at Screening, verified per site SOP
2. Able and willing to provide written informed consent
3. HIV-1/2 uninfected at Screening and Enrollment, per applicable algorithm in Appendix II and willing to receive HIV test results
4. Able and willing to provide adequate locator information, as defined in site SOP
5. Available to return for all study visits and willing to comply with study participation requirements

6. In general good health at Screening and Enrollment, as determined by the site Investigator of Record (IoR) or designee

7. At Screening, history of consensual RAI at least three times in the past three months and expecting to maintain at least this frequency of RAI during study participation per participant report

8. Willing to not take part in other research studies involving drugs, medical devices, genital or rectal products, or vaccines for the duration of study participation (including the time between Screening and Enrollment)

9. For individuals who can get pregnant (i.e., TGM with a female reproductive system): a negative pregnancy test at Screening and Enrollment

10. For individuals who can get pregnant: Per participant report at Enrollment, using an effective method of contraception for at least 30 days (inclusive) prior to Enrollment and intending to use an effective method for the duration of study participation; effective methods include:
    a) Hormonal methods
    b) Intrauterine device (IUD) inserted at least 30 days prior to Enrollment (but not past the maximum length of recommended usage according to package instructions)
    c) Sterilization (of participant or, if in a monogamous relationship, of partner, as defined in site SOPs)
    d) Abstinence from RVI for 90 days prior to Enrollment, and intention to abstain from RVI for the duration of study participation

5.3 Exclusion Criteria

Individuals who meet any of the following criteria will be excluded from the study:

1. At Screening:
    a) History of inflammatory bowel disease
    b) Current anorectal condition that would impede product placement or assessment of tolerability by participant report or exam

2. Anticipated use and/or unwillingness to abstain from using non-study rectally-administered medications and products during study participation, including personal lubricants containing nonoxynol-9 (N-9)

    Note: The use of non-study personal lubricants and usual pre-RAI douches that do not contain N-9 is permitted during study participation.

3. Known adverse reaction to any of the components of the study products

4. Participation in research studies involving drugs, medical devices, genital products, or vaccines within 30 days of the Enrollment Visit

5. Participation in research studies involving rectal products (ever)

6. Per participant report, use of post-exposure prophylaxis (PEP) for potential HIV exposure within the 3 months prior to Enrollment

7. In the 3 months prior to Enrollment, participant engagement in condomless RAI or RVI while not on PrEP with a partner who is HIV-positive and either not on ART or of unknown ART use status (by self-report)

8. In the month prior to Enrollment, participant engagement in condomless RAI or RVI while not on PrEP with a partner who is of unknown HIV status and unknown PrEP/ART use status (by self-report)

9. Non-therapeutic injection drug use in the 12 months prior to Enrollment

10. At either Screening or Enrollment, participant-reported symptoms and/or clinical or laboratory diagnosis of active anorectal or reproductive tract infection (RTI) requiring treatment per current WHO guidelines (http://www.who.int/hiv/pub/sti/pub6/en/), or symptomatic urinary tract infection (UTI). Infections requiring treatment include Neisseria gonorrhea (GC), Chlamydia trachomatis (CT), syphilis, active herpes simplex virus (HSV) lesions, anogenital sores or ulcers, or symptomatic genital warts, chancroid, pelvic inflammatory disease (PID), symptomatic bacterial vaginosis (BV), symptomatic vaginal candidiasis, and trichomoniasis.

    Note: Otherwise eligible participants with a symptomatic UTI or an STI/RTI requiring treatment per current WHO guidelines may be re-tested during the screening process and if treatment is completed and symptoms have resolved within the screening window the participant may be enrolled.

    Note: HSV-1 or HSV-2 seropositive diagnosis with no active lesions is permitted since treatment is not required.

11. For individuals who can get pregnant: Pregnant or breastfeeding at either Screening or Enrollment or planning to become pregnant during study participation

12. For individuals who can get pregnant: Last pregnancy outcome 90 days or less prior to Screening

13. Has any other condition that, in the opinion of the IoR/designee, would preclude informed consent, make study participation unsafe, complicate interpretation of study outcome data, or otherwise interfere with achieving study objectives.

5.4 Co-enrollment Guidelines

As indicated in Sections 5.2 and 5.3, participants must not take part in other research studies involving drugs, medical devices, genital or rectal products, or vaccines after the Screening Visit and while taking part in MTN-035 unless approved by the Protocol Safety Review Team (PSRT). Participation in the following types of studies may be allowed at the discretion of the IoR/designee:

- Participants may take part in ancillary studies if approved by MTN-035 PSRT
- Participants who become infected with HIV may take part in observational and/or interventional studies for HIV-positive persons

Should any participant report concurrent participation in contraindicated studies after enrolling in MTN-035, the IoR/designee will consult the PSRT regarding ongoing product use and other potential safety considerations associated with co-enrollment.

6 STUDY PRODUCT

6.1 Regimen

Participants will be randomized to one of six study product administration sequences (Sequence A-F). Each sequence will consist of three four-week periods of study product administration, with one week between each four-week period. The duration of product administration including the two washout periods is approximately 14 weeks.

Table 3: Study Product Regimen

Sequence	N	Period 1 (4 weeks)	Washout period (~1 week)	Period 2 (4 weeks)	Washout period (~1 week)	Period 3 (4 weeks)
A	35	Rectal insert	--	Rectal douche	--	Rectal suppository
B	35	Rectal douche	--	Rectal suppository	--	Rectal insert
C	35	Rectal suppository	--	Rectal insert	--	Rectal douche
D	35	Rectal insert	--	Rectal suppository	--	Rectal douche
E	35	Rectal douche	--	Rectal insert	--	Rectal suppository
F	35	Rectal suppository	--	Rectal douche	--	Rectal insert

6.2 Administration

Each participant will receive study product that includes placebo inserts, placebo suppositories, and placebo (water) douches for pericoital rectal administration. The products will be administered in order of the assigned sequence. It is recommended that participants insert one dose of the study product between 30 minutes and 3 hours prior to RAI, and that they not insert more than one dose in 24 hours. The study product should be administered following the participant's usual pre-RAI practices. If a participant does not engage in RAI in a given week, they will be asked to insert a dose of the product in the absence of RAI. Participants will self-administer the first dose of each product in the clinic to ensure correct administration and tolerability.

6.2.1 Placebo inserts and suppositories

Participants will be instructed to insert in the rectum a placebo insert or suppository prior to RAI. If a dose is missed, participants will be instructed to wait to take a dose at the next occurrence of RAI. Furthermore, if no RAI activity occurs at all in a given 7-day period, participants will be instructed to take a dose on the seventh day in the absence of RAI. Participants will be provided instructions on proper insertion of the insert and suppository.

6.2.2 Placebo rectal douche

Participants will be instructed to insert in the rectum a placebo douche prior to RAI. If a dose is missed, participants will be instructed to wait to take a dose at the next occurrence of RAI, or if no other RAI activity occurs in that 7-day period, to take a dose in the absence of RAI. Furthermore, if no RAI activity occurs at all in a given 7-day period, participants will be instructed to take a dose on the seventh day in the absence of RAI. Participants will be provided instructions on preparation and proper insertion of the douche.

6.3 Study Product Formulation

Study products should be stored at controlled room temperature 20° - 25°C (68° - 77°F) until dispensed for use. Excursions between 15° – 30°C (59° – 86°F) are allowed.

6.3.1 Placebo rectal insert

The placebo rectal insert is formulated into white to off-white uncoated solid dosage forms in a bullet shape. The insert contains the following inactive excipients: isomalt, xylitol, sodium CMC, povidone, hydroxypropyl methylcellulose, poloxamer 188, sodium stearyl fumarate and magnesium stearate. The insert is 1.5 cm (0.6 inches) long, 0.7 cm (0.28 inches) wide, 0.6 cm (0.23 inches) in height, and approximately 500 mg in weight. The rectal insert is manufactured under current Good Manufacturing Practice (cGMP) under CONRAD oversight. The insert is stored in a white induction-sealed HDPE bottle along with polyester coil and a desiccant at controlled room temperature (see temperature details above).

6.3.2 Placebo rectal douche

Participants will fill the enema bottle with approximately 120 mL of clean tap water or bottled water. The enema bottles used in this study are commercially available 125 mL enema bottles that meet US Pharmacopeia (USP) standards and contain no heavy metals, no phthalates, and are bisphenol A (BPA) free.

6.3.3 Placebo rectal suppository

The rectal suppository is approximately 3-3.8 cm (1.2-1.5 inches) long and 2 grams in weight. The placebo rectal suppository consists of a Witepsol® H5 (IOI Oleochemical) base and contains 15% diglyceride and not more than 1% monoglyceride content. The rectal suppository will be manufactured under current Good Manufacturing Practice (cGMP). The suppository is stored at controlled room temperature (see temperature details above).

6.4 Study Product Supply and Accountability

6.4.1 Study Product Supply

Placebo Rectal Insert

CONRAD will oversee the manufacture of the placebo inserts by CoreRx (Clearwater, FL)

Placebo Rectal Douche

Participants will be provided commercially available 125 mL enema bottles with pre-lubricated tips. Bottled water or clean tap water will be used for the rectal douches. Bottled water will be provided by the sites for participants who are unable to obtain clean tap water. Supplies and instructions for preparing the rectal douches will be provided to the study participants.

Placebo Rectal Suppository

The MTN will oversee the manufacture of the placebo suppositories by CoreRx (Clearwater, FL).

6.4.2 Study Product Accountability

The Clinical Research Site (CRS) Pharmacist of Record (PoR) is required to maintain complete records of the study products received and subsequently dispensed. All unused study products must be returned to the MTN LOC Pharmacist after the study is complete unless otherwise instructed by the MTN LOC Pharmacist.

6.5 Study Product Dispensing

Study products will be dispensed by the pharmacist to study staff upon receipt of a written prescription from an authorized prescriber. An authorized prescriber includes the IoR or a licensed clinician directly responsible to the IoR as noted on the DAIDS IoR Form. Study product will be dispensed at the beginning of each product use period including Enrollment (Day 0), Visit 4 (Day 35), and Visit 6 (Day 70). If additional supplies are needed between study visits, participants will be instructed to contact the study site.

6.6 Ancillary Study Supplies

Participants will be offered male condoms at all visits. The condoms will be made available in the clinic and will be dispensed by the clinic staff. Participants will be provided lubricant approved for use during the product administration periods to facilitate insertion. At all other visits, clinical staff will offer participants lubricant (not containing N-9) as per local standard of care.

6.7 Concomitant Medications and Practices

Enrolled study participants may use concomitant medications during study participation. All concomitant medications reported throughout the course of the study will be recorded in the study database. Concomitant medications include all prescription medications, over-the-counter preparations, vitamins, nutritional supplements, and herbal preparations.

Use of rectally-administered non-study medications and products is prohibited, including any products containing N-9.

Note: The use of non-study personal lubricants and usual pre-RAI douches that do not contain N-9 is permitted during study participation

7 STUDY PROCEDURES

An overview of the study visit and evaluations schedule is presented in Appendix I. Presented in this section is additional information on visit-specific study procedures. Detailed instructions to guide and standardize procedures as well as information regarding the study visit windows are provided in the MTN-035 SSP Manual available at http://www.mtnstopshiv.org/research/studies.

Figure 2: MTN-035 Study Visit Schedule

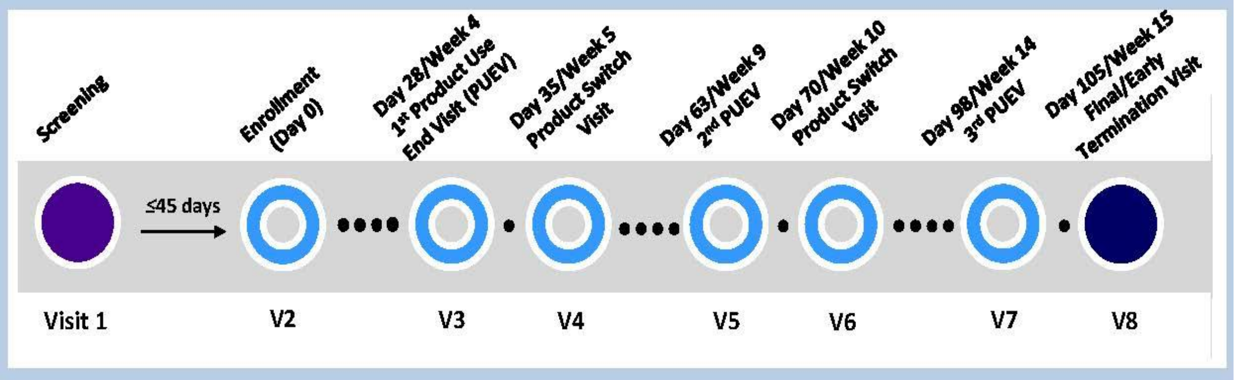

7.1 Pre-screening

As part of participant outreach and recruitment strategies, study staff can pre-screen potential study participants at either on-site or off-site locations. During these interactions, study staff may explain the study to potential participants and ascertain elements of presumptive eligibility (e.g., willingness to use the study products, willingness to adhere to the study requirements, etc.), to be confirmed at an on-site screening visit. Process information (e.g., number of potential participants contacted, number presumptively eligible) may be recorded and stored at the study site in the absence of written informed consent from potential participants, provided the information is collected in such a manner that it cannot be linked to participant identifiers, unless a waiver is granted from the local IRB/IEC. Procedures and documentation will comply with local IRB/IEC requirements.

7.2 Screening

A Screening Visit will take place up to 45 days prior to the Enrollment Visit (Day 0). Multiple visits may be conducted to complete all required screening procedures, if necessary. Written informed consent for Screening/Enrollment will be obtained before any screening procedures are initiated. For participants who do not meet the eligibility criteria, Screening will be discontinued once ineligibility is determined.

Note: If a participant is ineligible based upon a behavioral criterion, the behavioral eligibility assessment may be completed in its entirety, to avoid socially desirable reporting.

Table 4: Visit 1- Screening Visit

Visit 1-Screening Visit		
**Component**		**Procedures**
**Administrative and Regulatory**		• Obtain written informed consent<br>• Assess consent form comprehension<br>• Assign a unique Participant Identification (PTID) number<br>• Assess eligibility<br>• Collect demographic information<br>• Collect locator information<br>• Provide reimbursement<br>• Schedule next visit/contact*
**Behavioral/Counseling**		• HIV pre- and post-test counseling<br>• HIV/STI risk reduction counseling<br>• Protocol counseling
**Clinical**		• Collect medical history<br>• Collect concomitant medications<br>• Perform physical examination<br>• Perform rectal examination<br>• Disclose available test results<br>• Treat or prescribe treatment for RTI/UTI, or STIs*
**Laboratory**	**Pharyngeal**	• Nucleic acid amplification test (NAAT) for GC/CT
	**Urine**	• NAAT for GC/CT/Trichomonas<br>• Urine dipstick/culture*<br>• Pregnancy test ϕ
	**Blood**	• HIV-1/2 test<br>• Syphilis serology
	**Pelvic**	• NAAT for GC/CT and Trichomonas¤
	**Anorectal**	• NAAT for GC/CT<br>• HSV 1/2 detection* (at sites with capacity)
**Study Product/Supplies**		• Offer condoms<br>• Offer lubricant **

* If indicated and/or per local standard of care, ** Lubricant will be provided at product administration visits and as per local standard of care at other visits, ϕ Individuals who can get pregnant, ¤ Individuals with a vagina or neovagina

7.3 Enrollment (Day 0)

The Enrollment Visit occurs up to 45 days after the Screening Visit. Participants will be randomized to study product application sequence and begin using study product at the clinic during this visit.

Table 5: Visit 2- Enrollment Visit

<table>
<tr><th colspan="3">Visit 2- Enrollment Visit</th></tr>
<tr><th colspan="2">Component</th><th>Procedures</th></tr>
<tr><td colspan="2">Administrative and Regulatory</td><td>• Review informed consent/ confirm participant’s willingness to participate in study
• Assess and confirm eligibility
• Review/update locator information
• Provide reimbursement
• Schedule next visit/contact*
• Randomization</td></tr>
<tr><td colspan="2">Behavioral/Counseling</td><td>• HIV pre- and post-test counseling
• HIV/STI risk reduction counseling
• Protocol counseling
• Training in short message service (SMS)/instant message (IM) Reporting System
• Baseline behavioral assessment</td></tr>
<tr><td colspan="2">Clinical</td><td>• Review/update medical history
• Review/update concomitant medications
• Perform targeted physical examination
• Perform rectal examination
• Disclose available test results
• Treat or prescribe treatment for RTI/UTI, or STIs*</td></tr>
<tr><td rowspan="5">Laboratory</td><td>Pharyngeal</td><td>• NAAT for GC/CT*</td></tr>
<tr><td>Urine</td><td>• NAAT for GC/CT/Trichomonas*
• Urine dipstick/culture*
• Pregnancy test ϕ</td></tr>
<tr><td>Blood</td><td>• HIV-1/2 test
• Plasma for archive
• Syphilis serology*</td></tr>
<tr><td>Pelvic</td><td>• NAAT for GC/CT/Trichomonas*¤</td></tr>
<tr><td>Anorectal</td><td>• NAAT for GC/CT*
• HSV 1/2 detection* (at sites with capacity)</td></tr>
<tr><td colspan="2">Study Product/Supplies</td><td>• Provide study product and study product use counseling
• First study product application at the clinic
• Offer condoms
• Offer lubricant**</td></tr>
</table>

* If indicated and/or per local standard of care, ** Lubricant will be provided at product administration visits and as per local standard of care at other visits, ϕ Individuals who can get pregnant, ¤ Individuals with a vagina or neovagina

7.4 Follow-up Visits

7.4.1 Visits 3, 5 and 7 – First, Second and Third Product Use End Visits (PUEV)

Visit 3 – First PUEV should occur 4 weeks (approximately 28 days) after the Enrollment Visit. Visit 5 – Second PUEV should occur 4 weeks (approximately 28 days) after Visit 4 – Product Switch Visit. Visit 7 – Third PUEV should occur 4 weeks (approximately 28 days) after Visit 6 – Product Switch Visit. The PSRT must be consulted regarding progression into the next dosing period prior to the initiation of study product for Periods 2 and 3, for any participant who has unresolved pelvic, genital or anorectal AEs of Grade ≥3, and any other AE that in the opinion of the investigator would preclude the participant from continuing to the next product use period.

Participants will complete brief, weekly short SMS/IM assessments about their product use and RAI behaviors during each 4-week product use period. Behavioral evaluations will be completed at the end of each product use period at Visits 3, 5, and 7, including a brief in-depth interview (IDI) during the visit to review their SMS/IM reports.

Table 6: Visits 3, 5 and 7 – First, Second and Third PUEV

Visits 3, 5 and 7 – First, Second and Third PUEV		
**Component**		**Procedures**
**Administrative and Regulatory**		• Review/update locator information<br>• Provide reimbursement<br>• Schedule next visit/contact
**Behavioral/Counseling**		• HIV pre- and post-test counseling* (Required at Visit 7)<br>• HIV/STI risk reduction counseling* (Required at Visit 7)<br>• Protocol counseling<br>• Behavioral assessment<br>• Brief in-depth interview (IDI)
**Clinical**		• Review/update medical history<br>• Review/update concomitant medications<br>• Perform targeted physical examination*<br>• Perform rectal examination<br>• Disclose available test results<br>• Record/update AEs<br>• Treat or prescribe treatment for RTI/UTI, or STIs*
**Laboratory**	**Pharyngeal**	• NAAT for GC/CT* (Required at Visit 7)
	**Urine**	• Urine dipstick/culture*<br>• NAAT for GC/CT/Trichomonas* (Required at Visit 7)<br>• Pregnancy test* ϕ

Visits 3, 5 and 7 – First, Second and Third PUEV		
**Component**		**Procedures**
	**Blood**	• HIV-1/2 test* (Required at Visit 7)<br>• Syphilis serology* (Required at Visit 7)
	**Pelvic**	• NAAT for GC/CT/Trichomonas*¤ (Required at Visit 7)
	**Anorectal**	• NAAT for GC/CT* (Required at Visit 7)<br>• HSV 1/2 detection* (at sites with capacity)
**Study Product/Supplies**		• Offer condoms<br>• Offer lubricant**

* If indicated and/or per local standard of care, ** Lubricant will be provided at product administration visits and as per local standard of care at other visits, ϕ Individuals who can get pregnant, ¤ Individuals with a vagina or neovagina

7.4.2 Visits 4 and 6 – Product Switch Visits

The Visit 4 – Product Switch Visit should occur 1 week (approximately 7 days) after Visit 3 – First PUEV. The Visit 6 – Product Switch Visit should occur 1 week (approximately 7 days) after Visit 5 – Second PUEV.

Table 7: Visits 4 and 6 – Product Switch Visits

Visits 4 and 6 – Product Switch Visits		
**Component**		**Procedures**
**Administrative and Regulatory**		• Review/update locator information<br>• Provide reimbursement<br>• Schedule next visit/contact
**Behavioral/Counseling**		• HIV pre- and post-test counseling*<br>• HIV/STI risk reduction counseling*<br>• Protocol counseling
**Clinical**		• Review/update medical history<br>• Review/update concomitant medications<br>• Perform targeted physical examination*<br>• Perform rectal examination*<br>• Disclose available test results<br>• Record/update AEs<br>• Treat or prescribe treatment for RTI/UTI, or STIs*
**Laboratory**	**Pharyngeal**	• NAAT for GC/CT*
	**Urine**	• Urine dipstick/culture*<br>• NAAT for GC/CT/Trichomonas*<br>• Pregnancy test* ϕ
	**Blood**	• HIV-1/2 test*<br>• Syphilis serology*
	**Pelvic**	• NAAT for GC/CT/Trichomonas*¤
	**Anorectal**	• NAAT for GC/CT*<br>• HSV 1/2 detection* (at sites with capacity)

Visits 4 and 6 – Product Switch Visits	
**Component**	**Procedures**
**Study Product/Supplies**	• Provide study product and study product use counseling<br>• Study product application at the clinic<br>• Offer condoms<br>• Offer lubricant**

* If indicated and/or per local standard of care, ** Lubricant will be provided at product administration visits and as per local standard of care at other visits, ϕ Individuals who can get pregnant, ¤ Individuals with a vagina or neovagina

7.4.3 Visit 8 – Final/Early Termination Visit

The Visit 8 – Final Visit should occur 1 week (approximately 7 days) after Visit 7 – Third PUEV. This visit will serve as the participant's study termination.

During their Final or Early Termination Visit, participants will complete a conjoint analysis assessment to compare among the study products. Also, a subset of participants (approximately 10 per site) will be selected to complete an IDI during this visit to further explore participants' acceptability of the study products.

Table 8: Visit 8 – Final/Early Termination Visit

Visit 8 – Final/Early Termination Visit		
**Component**		**Procedures**
**Administrative and Regulatory**		• Review/update locator information<br>• Provide reimbursement
**Behavioral/Counseling**		• HIV pre- and post-test counseling*<br>• HIV/STI risk reduction counseling*<br>• Behavioral assessment<br>• IDI (subset)
**Clinical**		• Review/update medical history<br>• Review/update concomitant medications<br>• Perform targeted physical examination*<br>• Perform rectal examination*<br>• Disclose available test results<br>• Record/update AEs<br>• Treat or prescribe treatment for RTI/UTI, or STIs*
**Laboratory**	**Pharyngeal**	• NAAT for GC/CT*
	**Urine**	• Urine dipstick/culture*<br>• NAAT for GC/CT/Trichomonas*<br>• Pregnancy test ϕ
	**Blood**	• HIV-1/2 test*<br>• Syphilis serology*
	**Pelvic**	• NAAT for GC/CT/Trichomonas*¤
	**Anorectal**	• NAAT for GC/CT*<br>• HSV 1/2 detection* (at sites with capacity)

Visit 8 – Final/Early Termination Visit	
**Component**	**Procedures**
**Study Product/Supplies**	• Offer condoms<br>• Offer lubricant**

* If indicated and/or per local standard of care, ** Lubricant will be provided at product administration visits and as per local standard of care at other visits, ɸ Individuals who can get pregnant, ¤ Individuals with vagina or neovagina

7.5 Follow-up Procedures for Participants Who Permanently Discontinue Study Product

7.5.1 Participants Who Become Infected with HIV-1/2

If a participant tests positive for HIV-1/2 after the Enrollment Visit, s/he will be referred to local care and treatment services and may return to the research clinic for additional counseling and other support services, as needed. Continued study participation would be of no added benefit; thus follow-up visits will be discontinued and the participant will be considered terminated from the study. An Early Termination Visit will be conducted, if the participant is willing. Participants who are taking oral PrEP for HIV prevention while on study and seroconvert after randomization may be offered additional laboratory testing (such as HIV RNA and HIV drug resistance testing), as clinically indicated, per discussions between the site IoR and LC. Please reference the MTN-035 SSP Manual for additional details (www.mtnstopshiv.org/research/studies).

7.5.2 Participants Who Become Pregnant

Any participant who becomes pregnant will be referred to local health care services and may return to the research clinic for additional counseling, as needed. Continued study participation would be of no added benefit to the participant, thus follow-up visits and procedures will be discontinued and the participant will be considered terminated from the study. An Early Termination Visit will be conducted, if the participant is willing. See Section 9.7 for additional details.

7.5.3 Participants Who Permanently Discontinue Study Product for Other Reasons

For participants who permanently discontinue use of one of the study products for any clinician-initiated reason, the PSRT should be consulted regarding continued study participation and progression to other study products. For participants terminated from the study after PSRT consult, an Early Termination Visit will be conducted, if the participant is willing. See Section 9 for additional details regarding clinical follow-up of AEs unresolved at the time of study termination.

Participants who permanently discontinue use of one of the study products for any self-initiated reason during the first or second product use period will be allowed to continue in the study and progress to the other study product(s), if they are willing. Participants who permanently discontinue study product use for any self-initiated reason during the

third product use period will be terminated from the study, and an Early Termination Visit will be conducted, if the participant is willing. See Section 9.8 for additional details.

7.5.4 Interim Visits

Interim visits may be performed at any time during the study, for the following or other reasons:

- For administrative reasons, e.g., a participant may have questions for study staff, or may need to re-schedule a follow-up visit or to perform missed procedures.
- In response to AEs and/or SAEs. When interim contacts or visits are completed in response to participant reports of AEs and/or SAEs, study staff will assess the reported event clinically and provide or refer the participant to appropriate medical care (see also Section 9).
- For interim STI counseling and testing in response to STI symptoms.
- For interim HIV counseling and testing in response to participant report of symptoms consistent with acute infection or presumed exposure to HIV.
- To provide participants with the results of confirmatory HIV test results, per the algorithm in Appendix II.
- For other reasons at participant request, e.g., social harm, to get additional study product.

All interim contacts and visits will be documented in participants' study records.

7.6 Protocol Counseling: Adherence and Study Product Use Counseling

At the Enrollment and Product Switch Visits, participants will receive study product and study product use counseling appropriate to the visit. Study staff will document dispensation of study product and that the counseling was provided. Protocol adherence counseling will be provided to study participants upon enrollment into the study. Counseling will be provided in accordance with standard study methods. Counseling also will include reminders regarding concomitant medication and behavioral restrictions for the duration of the trial.

7.7 Clinical Evaluations and Procedures

<u>Physical Examination</u>

The physical examination will include the following assessments:

- General appearance
- Weight*
- Vital signs
  - Temperature
  - Pulse
  - Blood pressure
  - Respirations

- Height*
- Oral mucosa*
- Abdomen*
- Head, Eye, Ear, Nose and Throat (HEENT) Examination*
- Lymph nodes*
- Neck*
- Heart*
- Lungs*
- Extremities*
- Skin*
- Neurological*
- Pelvic Examination (*for individuals with a vagina or neovagina only*)**
  - Visual exam
  - Speculum exam
  - Bimanual exam
- Genital Examination (*for individuals with a penis or neopenis only*)**
  - Entire penile surface
    - Glans
    - Urethral meatus
    - Internal and external foreskin (if present)
    - Shaft
  - Scrotum
  - Inguinal lymph nodes

* = May be omitted after Screening Visit
** = May be omitted if not clinically indicated

Anorectal Examination

The anorectal examination may include the following:

- Visual exam
- Digital exam
- Anoscopy

Note: Detailed information regarding the pelvic, genital, and rectal examination, as well as associated procedures, can be found in the MTN-035 SSP Manual.

Additional clinical assessments may be performed at the discretion of the examining clinical staff in response to symptoms or illnesses present at the time of the exam.

7.8 Behavioral Evaluations

All participants will respond to computer assisted self-interviews (CASI) at the Enrollment Visit (Visit 2) and at the three PUEVs (Visits 3, 5 and 7), and to a conjoint analysis assessment at the Final Visit (Visit 8). The assessment done at enrollment will include, among other topics, questions on participants' prior experiences and comfort using rectal products, as well as douching or other rectal hygiene practices. The follow-up

assessments will explore reactions to each study product and administration method, including participants' perceptions of their partners' reactions. These assessments will allow investigators to identify product attributes likely to challenge and/or facilitate future sustained use when applied rectally by participants. Suggestions for product improvement will also be collected. The conjoint analysis assessment will compare between the three study products, including questions on participant preferences regarding the different formulations.

Participants will also complete a brief weekly report of product use via SMS/IM for each study product. A brief IDI is planned for all participants at the three PUEVs (Visits 3, 5 and 7). These IDIs will review and explore participants' responses to acceptability, tolerability, and adherence questions asked via SMS/IM during each product use period.

Based on Behavioral Research Working Group (BRWG) review of SMS/IM data, approximately 10 participants per site will be selected and invited to complete a longer IDI at the Final Visit (Visit 8). This IDI will include questions comparing user acceptability of the study products, user-centered suggestions for product design and delivery, acceptability and factors influencing product use, douching history and education, and experiences with the application methods, among other topics. Suggestions for product improvement will also be collected. The audio from the IDI will be recorded and transcribed for analysis. The interview notes, recording and transcript will be considered as source documentation.

Major components of both CASI and IDI assessments have been used successfully and validated in prior rectal microbicide trials (e.g., MTN-006, MTN-007, MTN-017) and are being implemented/planned as part of MTN-026, MTN-033 and MTN-037.

7.9 Laboratory Evaluations

Local Laboratory

The local laboratory will run the following, as indicated:

- Pharyngeal specimens at sites with capacity
    - NAAT for GC/CT
- Urine specimens
    - Human chorionic gonadotropin (hCG)
    - NAAT for GC/CT/Trichomonas
    - Dipstick/culture
- Blood specimens
    - HIV-1/2 testing, with confirmatory testing as needed
    - Syphilis serology
- Vaginal specimens (fluid)
    - NAAT for GC/CT/Trichomonas
- Anorectal specimens (rectal fluid) at sites with capacity
    - NAAT for GC/CT

– HSV 1/2 detection

Laboratory Center (LC)

- Blood specimens
  - Plasma archive
- Pharyngeal specimens from sites without local capacity
  - NAAT for GC/CT
- Anorectal specimens (rectal fluid) from sites without local capacity
  - NAAT for GC/CT

Once all required study analyses of collected specimens are complete, any remaining sample may be shipped to the MTN LC for use in study-related quality assurance and quality control testing. If study samples will be used for assay validation or proficiency testing that is not study related, all participant identifiers (PTID) will be removed from the samples prior to use.

7.10 Specimen Management

The study site will adhere to the standards of good clinical laboratory practice (https://www.niaid.nih.gov/sites/default/files/gclp.pdf), in accordance with current DAIDS Laboratory Requirements, MTN-035 SSP Manual (http://www.mtnstopshiv.org/research/studies_and site SOPs for proper collection, processing, labeling, transport, and storage of specimens to standardize procedures. Specimen collection, testing, and storage at the site laboratories will be documented when applicable using the Laboratory Data Management System (LDMS). In cases where laboratory results are not available due to administrative or laboratory error, the site is permitted to re-draw specimens. Further, as part of quality control, researchers may need to look at short pieces of non-coding repetitive DNA sequence (3-7 base pairs) from blood in the event of sample mix-up. This test will only let researchers know the number of times this short segment is repeated and not specific genes or specific sequences of base pairs. This sequence element does not contain any information about genes, therefore researchers will not be able to identify if participants are predisposed to specific diseases or any other genetic information based on this information. This test will be an important tool for distinguishing whether two samples collected at the same or different time points are likely from the same person. The test will only be used as part of a sample investigation with the knowledge of the site in situations where a known or suspected sample mix-up has occurred. No genetic testing (limited or genome-wide) is planned and all leftover samples will be destroyed after all testing related to this study is completed.

7.11 DAIDS Laboratory Oversight

All laboratories participating in DAIDS Sponsored and/or Funded Laboratories in Clinical Trials will adhere to the DAIDS Laboratory Policy.
(https://www.niaid.nih.gov/research/daids-clinical-research-policies-us-labs)

7.12 Biohazard Containment

As the acquisition of HIV and other blood-borne pathogens can occur through contact with contaminated needles, blood, and blood products, appropriate blood and secretion precautions will be employed by all personnel in the drawing of blood and shipping and handling of all specimens for this study as recommended by the CDC and National Institutes of Health (NIH). All biological specimens will be transported using packaging mandated by Code of Federal Regulations (CFR) 42 Part 72. All dangerous goods materials, including diagnostic specimens and infectious substances, must be transported according to instructions detailed in the International Air Transport Association (IATA) Dangerous Goods Regulations. Biohazardous waste will be contained according to institutional, transportation/carrier, and all other applicable regulations.

8 ASSESSMENT OF SAFETY

8.1 Safety Monitoring

The site IoR is responsible for continuous close safety monitoring of all study participants and for alerting the Protocol Team if unexpected concerns arise. A sub-group of the Protocol Team, including the Protocol Chair, DAIDS Medical Officer, representative(s) from CONRAD, and Protocol Safety Physicians will serve as the PSRT. The MTN Statistical Data Management Center (SDMC) prepares routine AE and clinical data reports for review by the PSRT, which meets via conference call approximately once per month or as needed throughout the period of study implementation to review safety data, discuss product use management, and address any potential safety concerns.

8.2 Clinical Data and Safety Review

A multi-tiered safety review process will be followed for the duration of this study. The study site investigators are responsible for the initial evaluation and reporting of safety information at the participant level and for alerting the PSRT if unexpected concerns arise. Participant safety is also monitored at the Network level through a series of routine reviews conducted by the SDMC and the PSRT. Additional reviews may be conducted at each of these levels as dictated by the occurrence of certain events.

MTN SDMC staff will review incoming safety data on an ongoing basis. Events identified as questionable, inconsistent, or unexplained will be queried for verification.

The PSRT will meet approximately every month via conference call to review clinical data reports generated by the MTN SDMC. The content, format and frequency of the clinical data reports will be agreed upon by the PSRT and the SDMC in advance of study implementation. In addition to the routine safety data reviews, the PSRT will convene on an ad hoc basis to make decisions regarding the handling of any significant safety concerns. If necessary, experts external to the MTN representing expertise in the fields

of microbicides, biostatistics, HIV acquisition and medical ethics may be invited to join the PSRT safety review. A recommendation to pause or stop the trial may be made by the PSRT at this time or at any such time that the team agrees that an unacceptable type and/or frequency of AEs has been observed.

The Study Monitoring Committee (SMC) will review participant safety data as part of its regular reviews (see Section 10.6), since no Data and Safety Monitoring Board oversight is planned for MTN-035. The SMC may recommend that the study proceed as designed, proceed with design modifications, or be discontinued. Members of the SMC will be independent investigators with no interest (financial or otherwise) in the outcomes of this study. If at any time a decision is made to discontinue enrollment and/or study product use in all participants, the Site IoR(s) will notify the responsible IRB(s)/IEC(s) expeditiously.

In addition to the safety monitoring, the MTN SMC will conduct interim reviews of study progress, including rates of participant accrual, retention, completion of primary and main secondary endpoint assessments, and study or lab issues. These reviews will take place approximately every 4-6 months, or as needed. At the time of these reviews, or at any other time, the SMC may recommend that the study proceed as designed, proceed with design modifications, or be discontinued.

8.3 Adverse Events Definitions and Reporting Requirements

8.3.1 Adverse Events

An AE is defined as any untoward medical occurrence in a clinical research participant administered an investigational product and which does not necessarily have a causal relationship with the investigational product. As such, an AE can be an unfavorable or unintended sign (including an abnormal laboratory finding, for example), symptom or disease temporally associated with the use of an investigational product, whether or not considered related to the product. This definition is applied to all groups beginning at the time of enrollment (i.e., once a participant is randomized) through to the termination visit. The term “investigational product” for this study refers to the placebo rectal insert, the placebo (water) rectal douche, and the placebo rectal suppository.

Study participants will be provided instructions for contacting the study site to report any untoward medical occurrences they may experience. In cases of potentially life-threatening events, participants will be instructed to seek immediate emergency care. Where feasible and medically appropriate, participants will be encouraged to seek evaluation where a study clinician is based, and to request that the clinician be contacted upon their arrival. Records from all non-study medical providers related to untoward medical occurrences will be obtained whenever possible and with appropriate permission of the participant, and required data elements will be captured in the study database. All participants reporting an untoward medical occurrence considered related to product use will be followed clinically until the occurrence resolves (returns to baseline) or stabilizes.

Study site staff will document in source documents and in the study database all AEs reported by or observed in enrolled study participants regardless of severity and presumed relationship to study product. AE severity will be graded per the DAIDS Table for Grading Adult and Pediatric Adverse Events, Corrected Version 2.1, July 2017 and/or Addenda 1, 2 and 3 (Female Genital [Dated November 2007], Male Genital [Dated November 2007] and Rectal [Clarification Dated May 2012] Grading Tables for Use in Microbicide Studies), available on the DAIDS Regulatory Support Center (RSC) website at http://rsc.tech-res.com/clinical-research-sites/safety-reporting/daids-grading-tables.

For individuals with a vagina or neovagina, please note:

- Asymptomatic BV and asymptomatic candida will not be reportable AEs, but will be captured on the STI CRF.

For individuals who can get pregnant, please note:

- Fetal losses (e.g., spontaneous abortions, spontaneous fetal deaths, stillbirths) will not be reported as AEs, but will be captured on the Pregnancy Outcome CRF.
- Untoward maternal conditions that either result in or result from fetal losses are reported as reproductive system AEs.

Fecal urgency, bloating, flatulence and bleeding associated with rectal procedures deemed to be within the range of what is normally expected will not be reportable as AEs. Bleeding of greater quantity or longer duration than what is typical, per clinician assessment, will be reportable as an AE.

8.3.2 Serious Adverse Events

An SAE will be defined by the Manual for Expedited Reporting of Adverse Events to DAIDS (Version 2.0, January 2010), which is available on the DAIDS RSC website at http://rsc.tech-res.com/clinical-research-sites/safety-reporting/manual, as an AE that:

- Results in death
- Is life-threatening
- Results in persistent or significant disability/incapacity
- Is a congenital anomaly/birth defect
- Requires inpatient hospitalization or prolongation of existing hospitalization

  Note: Per ICH SAE definition, hospitalization itself is not an AE, but is an outcome of the event. Thus, hospitalization in the absence of an AE is not regarded as an AE. The following are examples of hospitalization that are not considered to be AEs:
    - Protocol-specified admission (e.g., for procedure required by study protocol)
    - Admission for treatment of target disease of the study, or for pre-existing condition (unless it is a worsening or increase in frequency of hospital admissions as judged by the clinical investigator)
    - Diagnostic admission (e.g., for a work-up of an existing condition such as persistent pretreatment lab abnormality)
    - Administrative admission (e.g., for annual physical)
    - Social admission (e.g., placement for lack of place to sleep)

- Elective admission (e.g., for elective surgery)

Important medical events that may not result in death, be life-threatening, or require hospitalization may be considered an SAE when, based upon appropriate medical judgment, they may jeopardize the patient or subject and may require medical or surgical intervention to prevent one of the outcomes listed above.

8.3.3 Adverse Event Relationship to Study Product

Relatedness is an assessment made by a study clinician of whether or not the event is related to the study agent. Degrees of relatedness will be categorized according to current DAIDS-approved guidelines. Per the Manual for Expedited Reporting of Adverse Events to DAIDS (Version 2.0, January 2010), the relationship categories that will be used for this study are:

- *Related:* There is a reasonable possibility that the AE may be related to the study agent(s)
- *Not Related:* There is not a reasonable possibility that the AE is related to the study agent(s)

8.4 Pregnancy and Infant Outcomes

Pregnant persons are excluded from this study.

8.5 Regulatory Requirements

Information on all reported AEs will be included in reports to all applicable government and regulatory authorities. Site IoRs/designees will submit AEs and any relevant safety information in accordance with local regulatory requirements.

8.6 Social Harms Reporting

Although the study site will make every effort to protect participant privacy and confidentiality, it is possible that participants' involvement in the study could become known to others and that social harms may result. Social harms that are judged by the IoR/designee to be serious or unexpected will be reported to the PSRT and responsible site IRBs/IECs according to their individual requirements.

9 CLINICAL MANAGEMENT

Guidelines for clinical management and temporary product hold/permanent discontinuation of each study product are outlined in this section. In general, the IoR/designee has the discretion to hold use of the study product(s) at any time if s/he

feels that continued product use would be harmful to the participant or interfere with treatment deemed clinically necessary. Unless otherwise specified below, the IoR/designee must immediately consult the PSRT for further guidance on resuming use of the study product(s), continuing the hold temporarily, or progressing to permanent discontinuation of the study product(s). The IoR/designee will document all temporary product holds and permanent discontinuations on applicable CRFs.

Participants reporting any unresolved AEs considered related to product use at the time of study termination will be followed clinically until the occurrence resolves (returns to baseline) or stabilizes, and this information will be documented.

9.1 Grading System

AE severity grading is described in Section 8.3.

9.2 Dose Modification Instructions

No dose modifications will be undertaken in this study.

9.3 General Criteria for Permanent Discontinuation of Study Product

A participant will be permanently discontinued from all study product use by the IoR/designee for any of the following reasons:

- Acquisition of HIV infection; for those who acquire HIV, study product should be held beginning immediately upon recognition of the first positive/reactive HIV test
- Pregnancy
- Reported use of non-study rectal medications or products, including personal lubricants and usual pre-RAI douches containing N-9

  Note: Non-study personal lubricants and usual pre-RAI douches that do not contain N-9 are permitted

- Participant is unable or unwilling to comply with required study procedures, or otherwise might be put at undue risk to their safety and well-being by continuing product use, according to the judgment of the IoR/designee

A participant will be temporarily discontinued from all study product use by the IoR/designee and a PSRT query submitted for any of the following reasons:

- Acquisition of an anorectal STI

The IoR/designee must consult the PSRT once the temporary hold is initiated. Together, the IoR/designee and the PSRT will discuss resuming product use, continuing the temporary hold, or progressing to permanent discontinuation.

9.4 Follow-up in Response to Observed Adverse Events

Grade 1 or 2

In general, a participant who develops a Grade 1 or 2 AE as defined by the Division of AIDS Table for Grading the Severity of Adult and Pediatric Adverse Events, Corrected Version 2.1, July 2017 or Addenda 1, 2 and 3 (Female Genital [Dated November 2007], Male Genital [Dated November 2007] and Rectal [Clarification Dated May 2012] Grading Tables for Use in Microbicide Studies), regardless of relationship to study product, may continue product use. If the IoR/designee opts to discontinue study product, the PSRT must be notified.

Grade 3 or 4

For participants who develop a Grade 3 or 4 AE as defined by the Division of AIDS Table for Grading the Severity of Adult and Pediatric Adverse Events, Corrected Version 2.1, July 2017 or Addenda 1, 2 and 3 (Female Genital [Dated November 2007], Male Genital [Dated November 2007] and Rectal [Clarification Dated May 2012] Grading Tables for Use in Microbicide Studies), study product must be held and the IoR must consult with PSRT regarding continued use of that study product and progression to other study products.

9.5 Sexually Transmitted Infection/Reproductive Tract Infection

The IoR/designee must manage STI/RTI per current WHO guidelines, available at http://www.who.int/hiv/pub/sti/pub6/en/.

Per Section 9.3, acquisition of an anorectal STI will lead to a temporary product hold. Product use need not be held in the event of other types of STI/RTI requiring treatment, unless other temporary product hold/permanent discontinuation guidelines described elsewhere in Section 9 apply. Should the IoR/designee determine that a temporary product hold is warranted due to an STI or RTI, consultation with the PSRT is required.

9.6 HIV-1/2 Infection

Participants who test positive for HIV-1/2 must have study product held immediately by the IoR/designee. A participant who is confirmed to be HIV-1/2 positive during the course of the study will have study product discontinued, all follow-up visits will be discontinued and the participant will be considered terminated from the study, as per Section 7.5.1. Guidance regarding management and referral for participants confirmed to be HIV-positive is located in Section 13.10.

9.7 Pregnancy

Participants who can get pregnant will be encouraged to report all signs or symptoms of pregnancy to study staff. The IoR/designee will counsel any participant who becomes pregnant regarding possible risks to the fetus according to site SOPs. The IoR/designee

also will refer the participant to all applicable services; however, sites will not be responsible for paying for pregnancy-related care.

A participant who becomes pregnant during the study will have study product discontinued and will be terminated from the study, as per Section 7.5.2.

9.8 Criteria for Early Termination of Study Participation other than HIV-1/2 Infection and Pregnancy

Participants may voluntarily withdraw from the study for any reason at any time. The IoR/designee also may withdraw participants from the study to protect their safety and/or if they are unwilling or unable to comply with required study procedures, after consultation with the PSRT. Participants also may be withdrawn if NIAID, MTN, government or regulatory authorities including the Office for Human Research Protections (OHRP), or site IRBs/IECs terminate the study prior to its planned end date. Every reasonable effort is made to complete a final evaluation of participants who withdraw or are withdrawn from the study prior to completing follow-up (see details regarding the Early Termination Visit in Section 7.4.3). Study staff members will record the reason(s) for all withdrawals in participants' study records.

10 STATISTICAL CONSIDERATIONS

10.1 Overview and General Design

This is a multi-site, randomized-sequence, three-period, open label crossover study designed to compare acceptability, tolerability, safety, and adherence data within individuals who have been exposed to an insert, a douche and a suppository applied rectally and used prior to RAI. Approximately 210 HIV-uninfected participants will be randomized to receive one of six study product application sequences. In the first product use period, participants will use either the rectal insert, the rectal douche, or the rectal suppository prior to RAI during a 4-week-long period. After a 1-week washout period, they will use one of the other two study products prior to RAI during the second 4-week-long study product use period. After another 1-week washout period, they will use the remaining study product prior to RAI during the third 4-week-long study product use period.

10.2 Study Endpoints

Consistent with the primary study objective to evaluate the acceptability and tolerability of a placebo insert, a placebo douche, and a placebo suppository when applied rectally and used prior to RAI, the following endpoints will be assessed on a scale of 1 to 10 for each study product:

- Participant self-report of likelihood of product use if shown to be effective

Consistent with the primary study objective to evaluate adherence to a placebo insert, a placebo douche, and a placebo suppository when applied rectally and used prior to RAI over a 4-week-long period, the following endpoints will be assessed for each study product:

- Per participant report, percentage of occasions when study product was used as instructed (per protocol)

Consistent with the primary study objective to evaluate the safety of a placebo insert, a placebo douche, and a placebo suppository when applied rectally and used prior to RAI, the following endpoints will be assessed for each study product:

- Grade 2 or higher related AEs as defined by the Division of AIDS Table for Grading the Severity of Adult and Pediatric Adverse Events, Corrected Version 2.1, July 2017, and/or Addenda 1, 2 and 3 (Female Genital [Dated November 2007], Male Genital [Dated November 2007] and Rectal [Clarification Dated May 2012] Grading Tables for Use in Microbicide Studies)

10.3 Sample Size and Power

There is no control group for comparison in this study. The goal is not to compare each product to some standard but instead to estimate overall rates of acceptability, adherence and safety.

10.3.1 Primary Endpoints – Acceptability/Tolerability and Adherence

Based on previous studies we expect to observe a high rate of acceptability (>80%) which equates to ≤20% of participants reporting a low likelihood of using the product in the future (≤3 on the rating scale). Likewise, we expect to observe a high rate of participants reporting taking at least 90% of expected doses.

The table below shows the exact confidence intervals for acceptability and adherence, assuming α=0.05 based on the Binomial distribution and varying rates of acceptability and adherence. The power to rule out <50% and <70% acceptability or adherence given the rates is also shown. Calculations assume the outcomes are observed in 200 of the participants (5% loss to follow-up). For example, if the true acceptability or adherence rate is 90%, we have 100% power to rule out a true acceptability or adherence rate of 75% or lower. If we observe an 80% acceptability or adherence rate, the corresponding 95% confidence interval (CI) will be (74%, 85%).

Table 9: Exact 2-sided 95% Confidence Intervals Based on Various Rates of Acceptability or Adherence Endpoints

Acceptability rate / adherence rate	95% CI if rate is observed	Power to rule out 50% if rate is true	Power to rule out 70% if rate is true	Power to rule out 75% if rate is true
95%	(91, 98)	100%	100%	100%
90%	(85, 94)	100%	100%	100%
85%	(79, 90)	100%	100%	93%
80%	(74, 85)	100%	90%	35%

10.3.2 Primary Endpoint – Safety

To characterize the statistical properties of the safety endpoint, the table below presents the probability of observing zero, at least one, and two or more safety endpoints among 200 participants (allowing for the possibility of 5% dropout) assuming various "true" event rates. By assuming that we may only have safety evaluations on 200 participants, these calculations are conservative. However, safety analyses will include all enrolled participants.

Table 10: Analysis of Safety Event Frequency

Event Rate	P(0 events | n=200)	P(≥1 event | n=200)	P(≥5 events | n=200)	P(≥10 events | n=200)
0.1%	82%	18%	0%	0%
0.5%	37%	63%	0%	0%
1%	13%	87%	5%	0%
2%	2%	98%	37%	1%
5%	0%	100%	97%	55%

10.4 Randomization Procedures

Participants will be randomly assigned with the ratio 1:1:1:1:1:1 to one of six study product application sequences. The randomized assignments will be in blocks to keep the balance of equal allocation. The randomization scheme, including enrollment of replacement participants, will be generated and maintained by the MTN SDMC.

10.5 Participant Accrual and Retention

The accrual period is expected to require approximately 9-12 months. The study will enroll approximately 210 participants.

The target retention rate for each study visit is 95%. Therefore, once a participant is enrolled in the study, the study site will make every reasonable effort to retain the participant for the entire study duration so that the participant is evaluable.

10.6 Data and Safety Monitoring Procedures

No Data and Safety Monitoring Board oversight is planned for this study. The MTN Study Monitoring Committee (SMC) will conduct interim reviews of study progress, including

rates of participant accrual, retention, completion of primary endpoint assessments, and study or lab issues by arm of the study. These reviews will take place approximately every 4-6 months, or as needed. Reviews may also be conducted on an as needed basis. At the time of this review, or at any other time, the SMC may recommend that the study proceed as designed, proceed with design modifications, or be discontinued.

10.7 Data Analyses

10.7.1 Primary Analyses on Acceptability/Tolerability and Adherence Measures

All primary analyses will be based on data from the participants with data in all three periods of study (participants who complete each period of the study). Baseline values between these participants and those participants lost to follow-up will be described and used to interpret the generalizability of the results. Additionally, secondary analyses will analyze endpoints among all participants. The adherence and acceptability/tolerability endpoints (defined in Section 10.3.1) will be reported with 95% CI. The purpose of this protocol is not to compare the placebo products; therefore, we do not plan on doing comparisons between products in the primary analysis. Logistic regression models will be used to assess the order of product use (period effect) on acceptability/tolerance and adherence.

10.7.2 Primary Analysis on Safety Measures

All visits in which a participant has been exposed to the study product will be included in the primary analyses of safety. Secondary analyses including the washout period may also be performed. The number and the percentages of participants experiencing each safety endpoint (see Section 10.2) will be tabulated by study arm. Each participant will contribute once in each category (i.e., only for highest severity AE for each participant) for the calculation of event rates. Exact binomial confidence intervals will be calculated for each safety endpoint for each arm and Fisher's Exact test will be used to test for differences in event rates between the arms. Logistic and Poisson regression models will be used to assess the order of product use (period effect) on safety endpoints.

10.8 Missing Data

A retention rate of 95% is targeted. Based on previous MTN trials, minimal missing data is expected. If missing data rates are higher than anticipated (over 10%), sensitivity analyses will be conducted to assess the impact of missing data on trial inference. Assuming missing data are ignorable, we will use multiple imputation based on all available baseline predictors and available trial outcomes.

11 DATA HANDLING AND RECORDKEEPING

11.1 Data Management Responsibilities

Data collection tools will be developed by the MTN SDMC in conjunction with the protocol team. Quality control queries will be routinely generated for study site verification and resolution. As part of the study activation process, the study site must identify all CRFs to be used as source documents. Study CRF data will be entered and cleaned using the Medidata Rave EDC tool, a data management system compliant with the International Council on Harmonization (ICH) Good Clinical Practices (GCP) and US CFR guidelines for electronic data capture.

Audio files generated by the in-depth interviews will be transcribed and translated into English by the interviewers at Columbia University. Once the transcripts are cleaned and final, both the transcripts and the interview summary sheets will be electronically transferred to the central study team at the University of Pennsylvania using a secure File Transfer Protocol site, where they will be uploaded and managed using a qualitative software package. The qualitative data from MTN-035 will include the IDI transcripts and the summary sheets detailing major points of interest regarding acceptability and adherence from each interview. The research team at the University of Pennsylvania will manage all qualitative data for the study. A convention for file naming will be developed, and all data will be labeled according to this process. The research team will save all versions of the files on a secure, password-protected server.

11.2 Source Documents and Access to Source Data/Documents

All study sites will maintain source data/documents in accordance with Requirements for Source Documentation in DAIDS Funded and/or Sponsored Clinical Trials (https://www.niaid.nih.gov/sites/default/files/daids-sourcedocpolicy.pdf) and the relevant appendix regarding source documentation (https://www.niaid.nih.gov/sites/default/files/sourcedocappndx.pdf).

Each IoR/designee will maintain, and store securely, complete, accurate and current study records throughout the study. The IoR/designee will maintain all study documentation for a minimum of three years after submission of the site's final Financial Status Report to DAIDS, unless otherwise specified by DAIDS or the MTN LOC. These documents should be retained for a longer period, however, if required by the applicable regulatory requirements or by an agreement with the sponsor.

Study records must be maintained on site for the entire period of study implementation. Thereafter, instructions for record storage will be provided by DAIDS. No study records may be moved to an off-site location or destroyed prior to receiving approval from DAIDS.

11.3 Quality Control and Quality Assurance

The study site will conduct quality control and quality assurance procedures in accordance with current DAIDS policies. (https://www.niaid.nih.gov/sites/default/files/qmppolicy.pdf)

12 CLINICAL SITE OVERSIGHT

Formal clinical site oversight is not planned for this study. However, representatives of the MTN, including but not limited to the LOC, SDMC, LC and the behavioral team, will review study-related documentation and procedures during the study to ensure compliance with the study protocol, Good Manufacturing Practice (cGMP), Good Clinical Practices (GCP), good clinical laboratory practice guidelines (GCLP) and other applicable US, local and international regulatory requirements. Responsible parties will do the following:

- Assess study facilities, staffing and the quality of study conduct
- Verify proper collection and storage of biological specimens
- Verify proper storage, dispensing, and accountability of investigational study products

The IoR/designee will also allow inspection of study facilities and study-related documentation by authorized representatives of NIAID, NIH and/or contractors of NIH, OHRP, CONRAD, IRBs/IECs, and other local, US or international regulatory authorities. These entities may also assess implementation and documentation of internal site quality management procedures. A site visit log will be maintained at the study site to document all visits.

13 HUMAN SUBJECTS PROTECTIONS

Site investigators will make efforts to minimize risks to participants. Participants and study staff members will take part in a thorough informed consent process. Before beginning the study, the IoR/designee will have obtained IRB/IEC approval(s). The IoR/designee will permit audits by the NIH, OHRP, CONRAD, MTN LOC, IRBs/IECs, SDMC, and other local, US, or international regulatory authorities, or any of their appointed agents.

13.1 Institutional Review Boards/Ethics Committees

The participating institution is responsible for assuring that this protocol, the associated site-specific informed consent forms (ICFs), and study-related documents (such as

participant education and recruitment materials) are reviewed by an IRB/IEC responsible for oversight of research conducted at the study site. Any amendments to the protocol must be approved by the responsible IRBs/IECs prior to implementation.

After the initial review and approval, the responsible IRBs/IECs must review the study at least annually. Each IoR/designee will make safety and progress reports to the IRBs/IECs at least annually and within three months after study termination or completion. These reports will include the total number of participants enrolled in the study, the number of participants who completed the study, all changes in the research activity, and all unanticipated problems involving risks to human subjects or others. In addition, the results of all SMC reviews of the study will be provided to the IRBs/IECs. The study site will submit documentation of continuing review to the DAIDS Protocol Registration Office (PRO) in accordance with the DAIDS Protocol Registration Policy and Procedures Manual.

13.2 Protocol Registration

Prior to implementation of this protocol, and any subsequent full version amendments, the site must have the protocol and the protocol consent forms approved, as appropriate, by its local IRB/IEC and any other applicable regulatory entity (RE). Upon receiving final approval, the site will submit all required protocol registration documents to the DAIDS PRO at the RSC. The DAIDS PRO will review the submitted protocol registration packet to ensure that all the required documents have been received.

Site-specific ICFs *will not* be reviewed and approved by the DAIDS PRO, and the site will receive an Initial Registration Notification when the DAIDS PRO receives a complete registration packet. Receipt of an Initial Registration Notification indicates successful completion of the protocol registration process. A copy of the Initial Registration Notification should be retained in the site's regulatory files.

Upon receiving final IRB/IEC and any other applicable RE approval(s) for an amendment, the site should implement the amendment immediately. The site is required to submit an amendment registration packet to the DAIDS PRO at the RSC. The DAIDS PRO will review the submitted protocol registration packet to ensure that all the required documents have been received. Site-specific ICF(s) *will not* be reviewed and approved by the DAIDS PRO and the site will receive an Amendment Registration Notification when the DAIDS PRO receives a complete registration packet. A copy of the Amendment Registration Notification should be retained in the site's regulatory files.

For additional information on the protocol registration process and specific documents required for initial and amendment registrations, refer to the current version of the DAIDS Protocol Registration Manual.

13.3 Study Coordination

Study implementation will be directed by this protocol, which may not be amended without prior written approval from the Protocol Chair and DAIDS Medical Officer. Study implementation will also be guided by a common Study-Specific Procedures (SSP) Manual that provides further instructions and operational guidance on conducting study visits; data and forms processing; specimen collection, processing, and shipping; AE assessment, management and reporting; dispensing study products and documenting product accountability; and other study operations. Standardized study-specific training will be provided to the site by the MTN LOC, SDMC, LC and other designated members of the Protocol Team.

Close coordination between protocol team members is necessary to track study progress, respond to queries about proper study implementation, and address other issues in a timely manner. The PSRT will address issues related to study eligibility and AE management and reporting as needed to assure consistent case management and documentation. Rates of accrual, adherence, follow-up, and AE incidence will be monitored closely by the protocol team as well as the SMC.

13.4 Risk Benefit Statement

13.4.1 Risks

It is not expected that this trial will expose human subjects to unreasonable risk.

Phlebotomy
Phlebotomy may lead to excessive bleeding, discomfort, feelings of dizziness or faintness, bruising, swelling, venous thrombosis and/or infection.

Pharyngeal Swab
Pharyngeal (throat) swab collection often causes a momentary gagging reflex.

Rectal Exam and Fluid Collection
Insertion of a lubricated anoscope during the rectal examination will likely cause some discomfort or pressure in the rectum or anorectal area. Insertion of finger(s) in the rectum by a clinician during the rectal examination also will likely cause discomfort or pressure in the rectum or anorectal area. There is the risk of mild discomfort in addition to a slight risk of bleeding with the insertion of rectal swabs or sponges.

Vaginal Fluid Collection
Collection of vaginal fluid may cause discomfort or pressure in the vagina or genital area.

Rectal Douche
The main risk from administering a rectal douche is temporary discomfort. A hollow tube about the thickness of a pencil will be used to put approximately 120 mL of water into the rectum and flush it out again, along with any stool that is there. There is a risk of a

bloated/cramping feeling. The tube is small and will be lubricated, but it might cause some anal or rectal discomfort if the subject has any hemorrhoids or other painful conditions. There is also a remote possibility of rectal perforation associated with the use of rectal douche products.

Rectal products
Side effects observed with application of rectal products in previous research studies include: mild rectal fullness; incontinence or diarrhea; flatulence; mild abdominal pain; anal discharge and proctalgia.

Other Risks
Disclosure of HIV and STI status may cause worry, sadness or depression. Disclosure of HIV-positive status has been associated with depression, suicidal ideation, and denial as well as social isolation. Trained counselors will be available to help participants deal with these feelings.

Participation in clinical research includes the risks of loss of confidentiality and discomfort with the personal nature of questions when discussing sexual behaviors and other practices.

Participants at sites where local regulatory authorities require partner notification in response to diagnosed STI or HIV infection could have problems in their relationships. Participants could also have problems in their partner relationships associated with use of study product.

Site staff will make every effort to protect participant privacy while in the study. Although the study site will make every effort to protect participant privacy and confidentiality, it is possible that participants' involvement in the study could become known to others, and that social harms may result (i.e., because participants could become known as HIV-positive or at "high risk" for HIV infection). For example, participants could be treated unfairly or discriminated against, or could have problems being accepted by their families and/or communities.

13.4.2 Benefits

Participants in this study will experience no direct benefit. Participants and others may benefit in the future from information learned from this study. Specifically, information learned in this study may lead to the development of safe and effective interventions to prevent HIV acquisition and transmission. Participants also may appreciate the opportunity to contribute to the field of HIV prevention research.

Participants will receive HIV/STI risk reduction counseling, HIV and STI testing, and physical and rectal examinations. Participants may be provided or referred for STI treatment free of charge. In addition, STI testing and treatment may be offered and/or referrals may be provided for their partners. For other medical conditions identified as part of the study screening and/or follow-up procedures, participants will be referred to other

sources of care available in their community. Some participants may have the opportunity to access expedited treatment and benefit from decreased morbidity due to early diagnosis and treatment of abnormalities identified during tests, examinations and referrals.

13.5 Informed Consent Process

Written informed consent will be obtained from each study participant prior to screening. In obtaining and documenting informed consent, the IoR and their designees will comply with applicable local and US regulatory requirements and will adhere to GCP and to the ethical principles that have their origin in the Declaration of Helsinki. Study staff must document the informed consent process in accordance with the Requirements for Source Documentation in DAIDS Funded and/or Sponsored Clinical Trials (https://www.niaid.nih.gov/sites/default/files/daids-sourcedocpolicy.pdf). Participants will be offered copies of the ICF.

In addition to the ICF, the Protocol Team will work with study staff and community representatives to develop appropriate materials about the study and a standardized approach to the informed consent process to be implemented at the study site, which will be detailed in the SSP Manual.

The informed consent process will cover all elements of informed consent required by research regulations. In addition, the process will specifically address the following topics of importance to this study:

- The lack of efficacy of the placebo study products
- Randomization and the importance of participation in all study sequences to the success of the study
- The importance of adherence to the study visit and procedures schedule
- The potential medical risks of study participation (and what to do if such risks are experienced)
- The potential social harms associated with study participation (and what to do if such harms are experienced)
- The real yet limited benefits of study participation
- The distinction between research and clinical care
- The right to withdraw from the study at any time

13.6 Participant Confidentiality

All study procedures will be conducted in private, and every effort will be made to protect participant privacy and confidentiality to the extent possible. The study site will implement confidentiality protections that reflect the local study implementation plan and the input of study staff and community representatives to identify potential confidentiality issues and strategies to address them.

All study-related information will be stored securely. All participant information will be stored in locked areas with access limited to study staff. All laboratory specimens, study data, and administrative forms will be identified by coded number only to maintain participant confidentiality. All records that contain names or other personal identifiers, such as locator forms and informed consent forms, will be stored securely. All local databases will be secured with password-protected access systems. Forms, lists, logbooks, appointment books, and any other listings that link participants' identification numbers to identifying information will be stored in a locked file in an area with limited access. Participants' study information will not be released without their written permission, except as necessary for review, monitoring, and/or auditing by the following:

- Representatives of the US Federal Government, including the US OHRP, NIH and/or contractors of the NIH, and other local, US or international regulatory authorities
- Representatives of CONRAD, including study monitors
- Study staff
- Site IRBs/IECs

The MTN has a Certificate of Confidentiality from the US Department of Health and Human Services that is applicable to this study. This Certificate protects study staff from being compelled to disclose study-related information by any US Federal, State or local civil, criminal, administrative, legislative or other proceedings. It thus serves to protect the identity and privacy of study participants at US-based study sites.

13.7 Special Populations

13.7.1 Pregnant Women

Anyone who tests positive for pregnancy at the Screening or Enrollment Visit will not be eligible to participate in this study. Should a participant test positive for pregnancy after Enrollment, a product discontinuation will be implemented. Follow-up will be completed and data collected per Section 7.5.2. During the informed consent process, individuals who can get pregnant will be informed that the study products are not an effective method of contraception and the effects of the study products on a developing human fetus are unknown.

13.7.2 Children

The NIH has mandated that children be included in research trials when appropriate. This study meets “Justifications for Exclusion” criteria for younger children as set forth by the NIH. Specifically, “insufficient data are available in adults to judge potential risk in children” and “children should not be the initial group to be involved in research studies.” This study does not plan to enroll children under 18 years old.

13.8 Compensation

Pending IRB/IEC approval, participants will be compensated for time and effort in this study, and/or be reimbursed for travel to study visits and time away from work. Site specific reimbursement amounts will be specified in the site specific ICF.

13.9 Communicable Disease Reporting

Study staff will comply with local requirements to report communicable diseases, including HIV-1/2 identified among study participants to health authorities. Participants will be made aware of reporting requirements during the informed consent process.

13.10 Access to HIV-related Care

13.10.1 HIV Counseling and Testing

HIV test-related counseling will be provided to all potential study participants who consent to undergo HIV-1/2 screening to determine their eligibility for this study, and to all enrolled participants at each follow-up HIV-1/2 testing time point. Testing will be performed in accordance with the algorithm in Appendix II. Counseling will be provided in accordance with standard HIV counseling policies and methods at the site. In accordance with the policies of the NIH, participants must receive their HIV-1/2 test results to take part in this study.

13.10.2 Care for Participants Identified as HIV-Positive

An individual who has been identified as infected with HIV-1/2 will be managed or referred for management according to the local standard of care. Should a participant test positive for HIV after the Enrollment Visit, follow-up procedures will be performed as per Section 7.5.1. Please refer to Section 9.6 for further details.

13.11 Study Discontinuation

This study may be discontinued at any time by NIAID, the MTN, the OHRP, other government or regulatory authorities, or site IRBs/IECs.

14 PUBLICATION POLICY

DAIDS/NIAID and MTN policies will govern publication of the results of this study. Any presentation, abstract, or manuscript will be submitted by the investigator to the MTN Manuscript Review Committee, DAIDS, NIAID, NIMH, and CONRAD for review prior to submission.

15 APPENDICES

APPENDIX I: SCHEDULE OF STUDY VISITS AND EVALUATIONS

		Visit 1 SCR	Visit 2 ENR	Visits 3, 5 & 7 PUEV 1-3	Visits 4 & 6 Product Switch Visits	Visit 8 Final / Early Term Visit
**ADMINISTRATIVE AND REGULATORY**						
Informed consent (SCR/ENR)		X				
Assess consent form comprehension		X				
Review informed consent/Confirm participant willingness to be in study			X			
Assign PTID		X				
Collect demographic data		X				
Assess/confirm eligibility		X	X			
Collect/update locator information		X	X	X	X	X
Provide reimbursement		X	X	X	X	X
Schedule next study visit/contact		*	*	X	X	
Randomization			X			
**BEHAVIORAL/COUNSELING**						
HIV pre-/post-test and HIV/STI risk reduction counseling		X	X	X (Required at Visit 7)	*	*
Protocol counseling		X	X	X	X	
Training in SMS/IM Reporting System			X			
Behavioral assessment			X	X		X
In-depth interview (IDI)				X (brief IDI)		X (subset)
**CLINICAL**						
Collect/update medical history		X	X	X	X	X
Perform physical examination (Targeted after Screening Visit)		X	X	*	*	*
Perform rectal examination		X	X	X	*	*
Collect/update concomitant medications		X	X	X	X	X
Treat for UTI/RTI/STI or refer		*	*	*	*	*
Disclose available test results		X	X	X	X	X
Record/update AEs				X	X	X
**LABORATORY**						
**PHARYNGEAL**	NAAT for GC/CT	X	*	* (Required at Visit 7)	*	*
**URINE**	NAAT for GC/CT/Trichomonas	X	*	* (Required at Visit 7)	*	*
**URINE**	Urine dipstick/culture	*	*	*	*	*
**URINE**	Pregnancy test ϕ	X	X	*	*	X
**BLOOD**	Plasma for archive		X			
**BLOOD**	Syphilis serology	X	*	* (Required at Visit 7)	*	*

		Visit 1 SCR	Visit 2 ENR	Visits 3, 5 & 7 PUEV 1-3	Visits 4 & 6 Product Switch Visits	Visit 8 Final / Early Term Visit
	HIV-1/2 test	X	X	* (Required at Visit 7)	*	*
PELVIC	NAAT for GC/CT/Trichomonas ¤	X	*	* (Required at Visit 7)	*	*
ANORECTAL	HSV-1/2 detection (at sites with capacity)	*	*	*	*	*
	NAAT for GC/CT	X	*	* (Required at Visit 7)	*	*
**STUDY PRODUCT/SUPPLIES**						
Provision of study product and study product use counseling			X		X	
Study product application at the clinic			X		X	
Offer condoms		X	X	X	X	X
Offer lubricant		**	**	**	**	**

X =Required, * = As Indicated and/or per local standard of care, ϕ = Individuals who can get pregnant, ¤ = Individuals with a vagina or neovagina, ** Lubricant will be provided at product administration visits and as per local standard of care at other visits

APPENDIX II: ALGORITHM FOR HIV TESTING FOR SCREENING AND FOLLOW-UP

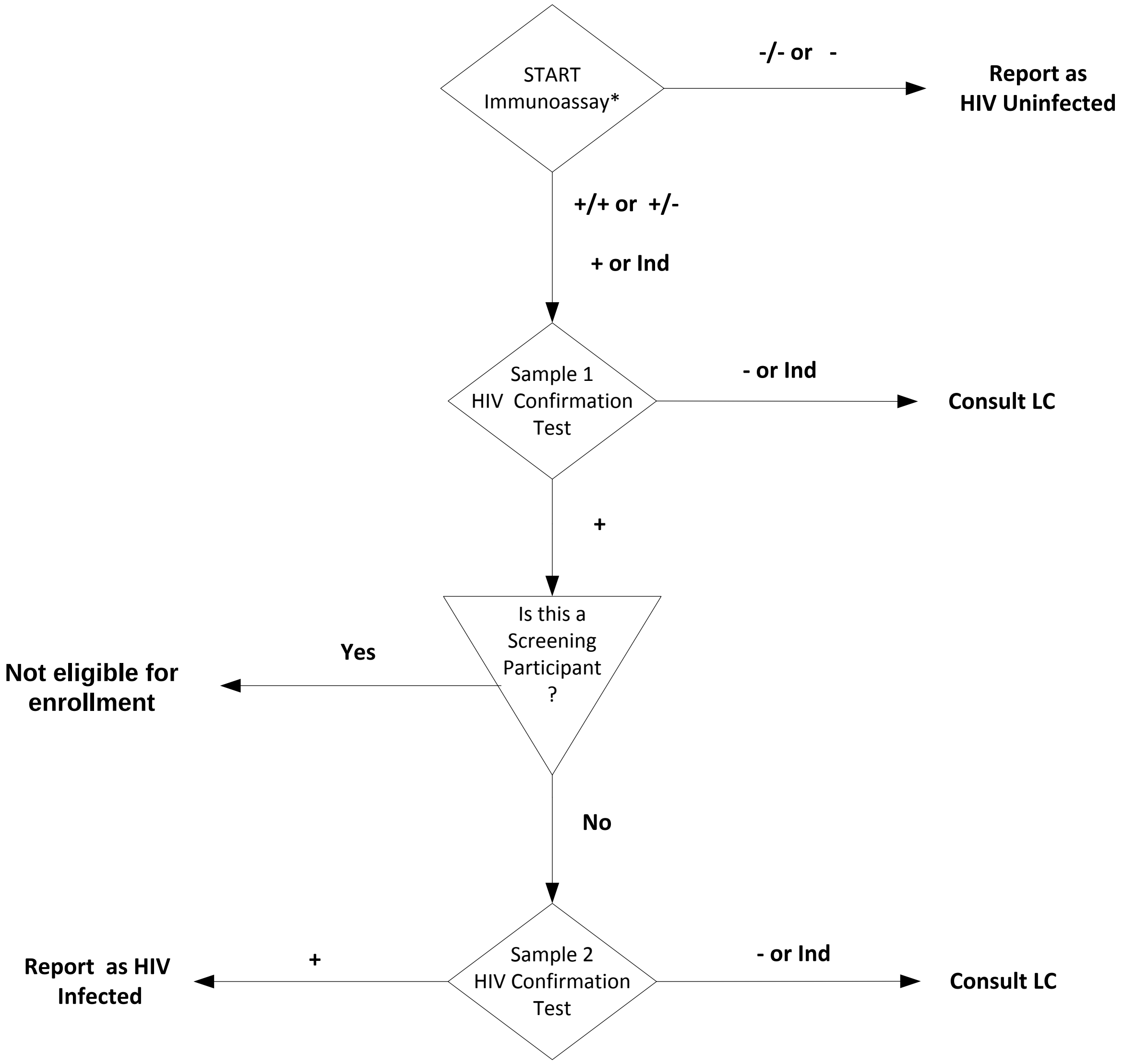

*CLIA certified labs may perform 1 rapid test
Ind: Indeterminate test results
LC: Laboratory Center

APPENDIX III: SAMPLE INFORMED CONSENT FORM (SCREENING AND ENROLLMENT)

DIVISION OF AIDS, NIAID, NIH

MTN-035

Acceptability, Tolerability, and Adherence of Three Rectal Microbicide Placebo Formulations among HIV Seronegative Cisgender Men, Transgender Men and Transgender Women Who Engage in Receptive Anal Intercourse

Version 1.0

June 15, 2018

PRINCIPAL INVESTIGATOR: ***[Site to insert]***
PHONE: ***[Site to insert]***
Short Title for the Study: Rectal Microbicide Acceptability, Tolerability, and Adherence

INFORMED CONSENT

IMPORTANT INFORMATION ABOUT THE RESEARCH STUDY

You are being asked to take part in this research study because you are a healthy, HIV-uninfected [***SITES TO INSERT PREFERRED DESCRIPTION OF STUDY POPULATION: cisgender man, transgender man (TGM), or transgender woman (TGW) / man who has sex with men (MSM) or transgender woman (TGW)***] aged 18 to 35 years old who engages in receptive anal sex (penis into rectum). Approximately 210 people will participate in this study at multiple sites across the world. The US National Institutes of Health (NIH) sponsors this Microbicide Trials Network (MTN) study. At this site, the person in charge of this study is ***[INSERT NAME OF PRINCIPAL INVESTIGATOR]***.

Important things you should know:

- The three study products in this clinical trial are a rectal douche, a rectal suppository, and a rectal insert. All three are placebo study products.
- The purposes of the study are:
  - To find out if [***SITES TO INSERT PREFERRED DESCRIPTION OF STUDY POPULATION: cisgender men, TGM, and TGW / MSM and TGW***] would accept and tolerate three different ways to potentially deliver [***SITES TO INSERT PREFERRED DESCRIPTION: anti-HIV / antiretroviral***] drugs into the rectum when used before receptive anal sex.
  - To understand whether [***SITES TO INSERT PREFERRED DESCRIPTION OF STUDY POPULATION: cisgender men, TGM, and TGW / MSM and TGW***] would actually use these three rectal products as part of their usual practices around receptive anal sex.
  - To evaluate if each study product is safe when applied rectally and used before receptive anal sex by [***SITES TO INSERT PREFERRED DESCRIPTION OF STUDY POPULATION: cisgender men, TGM, and TGW / MSM and TGW***].

- If you qualify and choose to participate, you will be asked to use the three rectal products before receptive anal sex, each for one month (4 weeks). The order in which you will use the three products will be decided by chance *[**SITES TO INSERT PREFERRED DESCRIPTION OF 'RANDOMIZATION'**]*. Neither you nor the study staff can decide the order in which you use the products.
- Once enrolled, you will be asked to attend 8 clinic visits here at this study clinic, including the Screening Visit which is taking place today. You will come to the clinic every four and five weeks. The total length of your participation in the study will be approximately three and a half months.
- At some of the clinic visits, the following will occur:
    - A physical and/or rectal exam will be performed;
    - Blood will be obtained to test for HIV and/or other sexually transmitted infections (STI);
    - Urine will be collected to test for STIs and (if applicable) pregnancy;
    - Rectal, throat, and (if applicable) vaginal swabs will be collected to test for STIs;
    - You will be asked to complete a short interview. You may also be selected to complete a longer interview at the final visit.
- Some risks or discomforts from previously tested rectal products include:
    - Abdominal bloating, feeling full, or a sense of abdominal pressure and/or pain;
    - Sudden, almost uncontrollable, need to relieve the bowels;
    - Diarrhea (loose, frequent stools);
    - Passing gas from the intestinal tract;
    - Feeling a constant need to pass stools, despite an empty bowel;
    - Anal discharge.
- You may not experience any direct benefit from participation in this study, but you may learn more about HIV and other diseases and ways to protect yourself from acquiring HIV or other STIs. You will also have physical and rectal exams, receive HIV and STI counseling, and get referrals for other care if needed.
- Taking part in this research project is voluntary. You do not have to participate and you can stop at any time. This will not affect the service you get at this clinic or clinics in surrounding areas.
- If you decide not to join this study, there are other currently available methods to prevent sexually transmitted HIV: condom use during sex and/or the use of daily oral Truvada® for pre-exposure prophylaxis (PrEP) or post-exposure prophylaxis (PEP). Study staff can provide you with additional information about PrEP and PEP if you are interested. You may also join another study, if we have one and if you meet its requirements.

Please take time to read this entire form and ask questions before you decide if you want to join this study. Once you read and understand the study and its requirements, you can decide if you want to join. If you do decide to take part in the study, you will sign your name on this form. A copy of this document will be offered to you. Signing this consent form does not mean you will be able to join the study. You must first complete the screening tests and exams to see if you are eligible.

It is important to know that for this study these three rectal products will not contain any active drug. This means the products we will ask you to use will not protect you from acquiring HIV or any other STIs. Therefore, you should continue using HIV and STI prevention methods while in the study.

Who will be in this research study and what will I be asked to do if I join?

Approximately 210 participants will be enrolled in the MTN-035 study across 7 sites, with approximately 30 participants enrolled at each site.

If you enroll in the study, you will be asked to use the three rectal products before receptive anal sex, each for one month (4 weeks). The douche in this study will involve spraying water from the nozzle of a bottle into your rectum. The suppository is a small, cone-shaped object that you put in your rectum and dissolves quickly. An insert is a small, bullet-shaped object that you put in your rectum and dissolves quickly.

If it seems like you can join this study based on the tests done today, you will be asked to come back for an Enrollment Visit no later than 45 days from today. At your Enrollment Visit, you will begin using the first of three rectal products – douche, suppository or insert – depending on which product use sequence you have been assigned to.

Study Visit Schedule

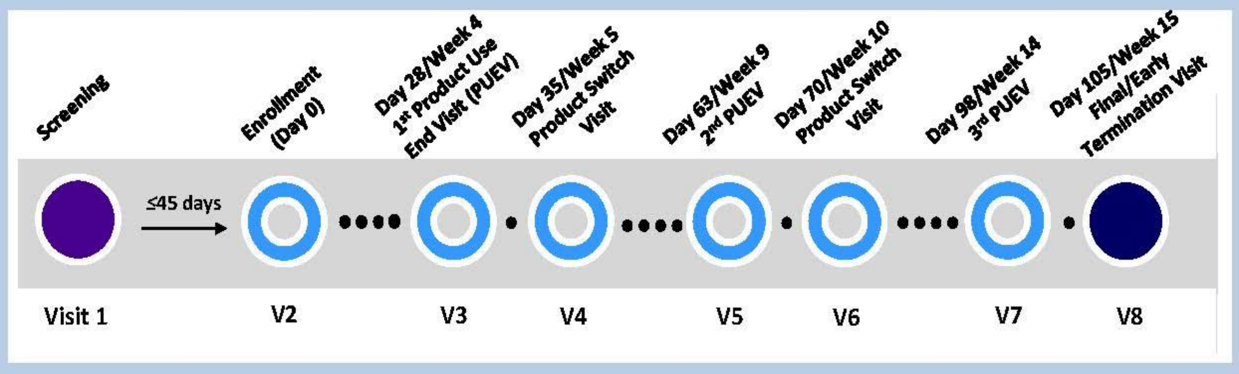

What will happen during study visits?

Screening Visit:

The procedures done today will take about ***[SITES TO INSERT TIME]***. Multiple visits may be conducted to complete all required screening procedures.

You will:

- Answer questions to confirm you are able and willing to join the study.
- Answer questions about where you live and other questions about you, your health (including what medications you are taking), and your sexual practices.
- Provide study staff your contact information (i.e. about how we can locate you).
- Talk with study staff about STIs, HIV, HIV/STI testing, and ways to avoid HIV and other infections passed through sex, including the use of oral Truvada® for PrEP.
- Talk with study staff about the requirements of the study including, but not limited to:
    - Abstaining from using non-study rectally-administered medications or products during your participation in the study, except for personal lubricants and usual pre-RAI douches that do not contain nonoxynol-9 (N-9).
- Have a physical exam.
- Have a rectal exam. Rectal fluid will be collected using a cotton swab (like a Q-tip or earbud) to test for STIs.
    - Study staff may insert a short (10 cm or 3-4 inches) hollow tube called an anoscope inside your rectum as part of the rectal exam and rectal fluid collection. Study staff

will insert their finger(s) inside your rectum as part of the rectal exam. You may feel some discomfort or pressure in your rectum or anus from the staff person's finger(s) and/or anoscope. There is a slight risk of bleeding with the insertion of rectal swabs or sponges.

- Have your urine tested for STIs and other infections.
- [***For individuals who can get pregnant***] Have your urine tested for pregnancy.
    - If you are pregnant you cannot join this study.
    - Staff will discuss with you ways to avoid getting pregnant.
- Have a pharyngeal (throat) swab collected to test for STIs.
- ***[For individuals with a vagina or neovagina]*** Have some fluids collected from your vagina using a swab to test for STIs.
- Provide a blood sample of about 10 ml ***[SITES TO MODIFY AMOUNT IF NEEDED]***:
    - To test for STIs, including HIV and syphilis.
        - You will be told your test results as soon as they are available. You will talk with study staff about the meaning of your results, how you feel about them, and learn about ways to prevent HIV and other STIs. Sometimes HIV tests are not clearly positive, but also not clearly negative. In that case, we will do more tests until we can confirm your status. To participate in the study, you must receive your HIV test results. If the test shows you have HIV, you cannot join the study. We will refer you to available sources of medical care and other services you may need. The study staff will tell you about other studies you may be eligible for, if any.
- Be given treatment or a referral for treatment of STIs, if needed.
- Be informed about other services, if needed.
- Be given the results of your tests, when available. It is expected that all your results will be available by ***[SITES TO SPECIFY TIMEFRAME]***.
- Be given male condoms, if you need them.
- ***[SITES TO SPECIFY IF PARTICIPANTS MAY BE OFFERED LUBRICANT PER LOCAL STANDARD OF CARE]***
- Be reimbursed for your visit.
- Schedule your next visit to enroll in the study, if you are willing and eligible.

If you decide not to join this study, blood and other samples collected at this visit will not be kept or used for any tests other than those listed above.

Enrollment Visit:

Your <u>Enrollment Visit</u> (the visit where you enter the study) will take place up to 45 days after your Screening Visit. This visit will take about ***[SITES TO INSERT TIME]***.

You will:

- Answer questions to confirm you are able and willing to join the study.
- Update study staff with your contact information (about where you live and how we can contact you).
- Be assigned the order in which you will use the three study products.
- Talk with study staff about the following:
    - The instructions and procedures of the study and how to follow the study rules, including use of non-study rectal medications or products.
    - STIs, HIV, HIV/STI testing, and ways to avoid HIV and other STIs.

- Discuss any health or medical problems you may have had in the past or since your last visit (including any medications you are taking).
- Be asked some questions about your experience and comfort using rectal products and engaging in rectal hygiene practices such as douching, among other things. These questions will be asked privately via computer.
- Be asked to respond to weekly electronic messages related to your use of the study products during each product use period.
    - You will receive instructions and training on how to receive and respond to these messages at the Enrollment Visit.
- Provide a blood sample of about 15 ml ***[SITES TO MODIFY AMOUNT IF NEEDED]***:
    - In case there's a question about your test results at a later time (about 10 ml of blood may be left unused if there is no question about your test results).
    - To test your blood for HIV.
- Have a physical exam.
- Have a rectal exam.
- [***For individuals who can get pregnant***] Have a urine test for pregnancy and discuss with study staff ways to avoid getting pregnant.
- Receive the first of three rectal products (douche, suppository, or insert) that you will use during the study, along with instructions on how to use and store your assigned product.
    - You will use your assigned study product once at the clinic under the supervision of study staff to ensure you can tolerate using the product and will be able to use it as instructed during the study.
- Be given test results, if available.
- Be given male condoms, if you need them.
- Be given lubricant to assist product insertion.
- Be reimbursed for your visit.
- Schedule your next visit, if applicable.

Product Use End Visits (PUEV) – Visits 3, 5, and 7:

Your PUEVs will take place approximately one month (4 weeks) after you begin using each of the three study products, with the first PUEV taking place approximately one month after the Enrollment Visit. These visits will take between ***[SITES TO SPECIFY TIMEFRAME]*** to complete.

You will:

- Update study staff with your contact information.
- Review the instructions and procedures of the study and how to follow the study rules, including use of non-study rectal medications or products.
- Discuss any health or medical problems you may have had since your last visit (including any medications you are taking).
- Discuss any problems that you may be experiencing as a result of using the study product, or as a result of procedures performed during the study.
- Be asked some questions about your experience using the study product. These questions will be asked privately via computer.
- Be asked to take part in a brief interview (~15 minutes) about your responses to the electronic messages during the previous month of product use. This interview will be conducted by phone or via videoconference. This interview will be audio-recorded, but your responses will be kept private and confidential.

- Have a rectal exam.
- Be given test results, if testing was done.
- Be given male condoms, if you need them.
- ***[SITES TO SPECIFY IF PARTICIPANTS MAY BE OFFERED LUBRICANT PER LOCAL STANDARD OF CARE]***
- Be reimbursed for your visit.
- Schedule your next visit or contact.

On your third PUEV (Visit 7), you will also:

- Talk with study staff about STIs, HIV, HIV/STI testing, and ways to avoid HIV and other STIs.
- Provide a blood sample of about 10 ml ***[SITES TO MODIFY AMOUNT IF NEEDED]*** to test your blood for STIs, including HIV and syphilis.
- Have your urine tested for STIs and other infections.
- Have a pharyngeal (throat) swab collected to test for STIs.
- Provide a rectal fluid sample. This will be collected using a swab to test for STIs.
- [***For individuals with vagina or neovagina***] Provide a vaginal fluid sample. This will be collected using a swab to test for STIs.

Product Switch Visits – Visits 4 and 6:

Your Product Switch Visits, Visits 4 and 6, will take place approximately one week after your first and second PUEVs, Visits 3 and 5, respectively. These visits will take between ***[SITES TO SPECIFY TIMEFRAME]*** to complete.

You will:

- Update study staff with your contact information.
- Review the instructions and procedures of the study and how to follow the study rules, including use of non-study rectal medications or products.
- Discuss any health or medical problems you may have had since your last visit (including any medications you are taking).
- Discuss any problems that you may be experiencing as a result of using the study product, or as a result of procedures performed during the study.
- Receive the rectal product assigned for your next product use period, along with instructions on how to use and store your assigned product.
    - You will use your assigned study product once at the clinic under the supervision of study staff to ensure you can tolerate using the product and will be able to use it as instructed during the study.
- Be given test results, if testing was done.
- Be given male condoms, if you need them.
- Be given lubricant to assist product insertion.
- Be reimbursed for your visit.
- Schedule your next visit or contact.

Final/Early Termination Visit – Visit 8:

Your Final/Early Termination Visit will take place approximately one week after your third PUEV, Visit 7. This visit will take approximately ***[SITES TO SPECIFY TIMEFRAME]*** to complete.

You will:

- Update study staff with your contact information.
- Discuss any health or medical problems you may have had since your last visit (including what medications you are taking).
- Discuss any problems that you may be experiencing as a result of using the study product, or as a result of procedures performed during the study.
- Be asked some questions to compare your experiences using the three study products. These questions will be asked privately via computer.
    - You may also be asked to take part in an in-depth interview (~45 minutes) about your experiences using the three study products, including questions about product design, application methods, product use influences, douching practices, and suggestions for product improvement. This interview will be conducted by phone or via videoconference. This interview will be audio-recorded, but your responses will be kept private and confidential.
- [***For individuals who can get pregnant***] Have a urine test for pregnancy and discuss with study staff ways to avoid getting pregnant.
- Be reimbursed for your visit.
- Be given test results, if available.
- Be given male condoms, if you need them.
- ***[SITES TO SPECIFY IF PARTICIPANTS MAY BE OFFERED LUBRICANT PER LOCAL STANDARD OF CARE]***

It is important that you remember that at any time during the study, study staff can answer any questions you may have about the procedures mentioned above or any other aspect of this study.

Other Procedures:
In addition to the procedures listed above, it is possible that study clinicians may need to perform additional tests during any of the study visits, if necessary (e.g., if you report having symptoms of a urinary, genital, or other infection and/or other issues). These tests might include the following:

- Physical exam
- Rectal exam
- Test pharyngeal (throat), rectal and/or pelvic samples for STIs
- Test your urine for STIs or other infections
- [***For individuals who can get pregnant***] Test your urine for pregnancy
- Test your blood for HIV and/or STIs
- Give you treatment or refer you for treatment of STIs or other issues, if needed.

You may need to provide additional samples if any of the study visit procedures need to be repeated due to issues with sample processing, testing and/or shipping. Additional testing may be performed as part of quality control.

It may also be necessary for additional visits to be conducted for unforeseen events. For example, you may be asked to come to the clinic for repeat or additional testing to ensure that the study products continue to be safe for you to use. Or you may choose to come for an additional visit for any unforeseen issues, questions, or counseling.

Some of your specimens collected in this study may be shipped and stored outside your country so that study-related testing can be performed. There might be a small amount of urine, blood, or vaginal and rectal fluid left over after we have done all of the study related testing, including testing

for quality assurance and control. These leftover samples will not be stored or used for any future testing, and they will be destroyed after all testing related to this study is completed.

What are the possible risks, side effects, and discomforts of this research study?

Risks from use of rectal products

The use of any rectal product can cause some side effects. We do not yet know all the side effects of the three study products. Previously tested rectal products have been associated with:

- Abdominal bloating, feeling full, or a sense of abdominal pressure and/or pain
- A sudden, almost uncontrollable, need to relieve the bowels
- Diarrhea (loose, frequent stools)
- Passing gas from the intestinal tract
- Feeling a constant need to pass stools, despite an empty bowel
- Anal discharge
- Proctalgia (anal pain)

Risks from phlebotomy (blood tests)

- You may feel discomfort
- You may feel dizzy or faint
- You may have a bruise, swelling, small clot, or infection where the needle goes in your arm
- You may have excessive bleeding

Risks of throat swab

- A pharyngeal (throat) swab often causes a momentary gagging reflex (feeling like you want to vomit).

Risks of rectal douching

- The main risk from rectal douching is temporary discomfort. An enema bottle will be used to administer about 120-125 mL (i.e., 4 ounces or 8 tablespoons) of water into the rectum.
- You may experience some mild discomfort and a bloated or “crampy” feeling.
- If you have any hemorrhoids or other painful conditions, you might feel anal or rectal discomfort.
- Some air may be pumped into the rectum as well, causing flatulence (feeling like you need to pass gas).
- There is a remote possibility of rectal perforation.

Risks of rectal exams

- During rectal exams and collection of rectal fluid samples, insertion of the study staff’s finger(s) and/or insertion of a lubricated anoscope will likely cause mild discomfort.

Risks of rectal swab/sponge insertion

- Insertion of rectal swabs or sponges may cause mild discomfort, in addition to a slight risk of bleeding.

(*For individuals with a vagina or neovagina*) Risks of vaginal swab

- During vaginal fluid collection, you may feel discomfort or pressure in your vagina or genital area.

Other Possible Risks

- You may become embarrassed and/or worried when discussing your sexual practices, ways to protect against HIV and other infections passed through sex, and your test results.
- You may feel anxious while waiting for your test results, and after receiving them. Trained study counselors will help you deal with any feelings or questions you have.
- Finding out your HIV status could also cause problems between you and your partner(s). If you have any problems, study counselors will talk with you and/or your partner to try to help resolve them.
- Disclosure of your HIV and STI status may cause worry, sadness or depression. Disclosure of HIV-positive status has been associated with depression, suicidal ideation, and denial as well as social isolation.
- It is possible that others may learn of your participation here and, because of this, may treat you unfairly or discriminate against you. For example, you could have problems getting or keeping a job, or being accepted by your family or community.
- The interviews that take place at some of your clinic visits will be computer-administered and questions of a personal nature may be asked. Responding to these questions may make you uncomfortable. You may choose not to answer any question that makes you uncomfortable.

We will make every effort to protect your privacy and confidentiality during the study visits. Your visits will take place in private. Reports via computer or text messages will be stored in computers that are password-protected and will not include personal information that could identify or link information to you; only your study ID number will be recorded. You will be shown how to erase the text message sessions from your mobile phone by study staff. However, as with all text messages sent from and received on your phone, it is possible that others may see your personal messages. Precautions have been taken to ensure that questions asked via text message are vague and will not directly convey information about your participation in this research study. There is a possibility that you may exceed your cell phone plan limit and have to pay for that.

When staff talk with you about how and when you used the study products they will audio record the discussion using a digital audio recorder. These audio files will be reviewed and analyzed by another person who is outside of the research site and does not know you or have your personal information. In addition, if you agree and are selected to participate in the in-depth interviews, these will be audio recorded. The audio files will be put into writing by the person interviewing you or by another person who does not know you and does not have your personal information. Any names that might be mentioned during the interview will NOT be retained. Instead a generic description will be used in the records (i.e., if you refer to a friend's name, "FRIEND1" will be noted).

Your audio recordings and any other information that links you to the research materials will be kept in a secure location that will be accessed only by members of the MTN-035 study team for the purposes of this research. *[Sites to modify with their site-specific source documentation storage duration requirements if required by their IRBs/IECs:* The audio recordings, notes, and transcripts from these materials will be kept for at least three years after the final study report is submitted to the study sponsors.]

What are possible benefits from taking part in this study?

- There are no direct benefits for taking part in this study, but you or others may have future benefit from information learned in this study. You may also learn more about HIV and other diseases and ways to protect yourself from acquiring HIV or other STIs.
- You will have physical and rectal exams. If these exams show that you might have any health problems, you will be told about medical care and other services available to you. This will be available to you even if you do not enroll in this study. This study cannot provide you with general medical care, but study staff will refer you to other available sources of care, if needed.
- You will get counseling and testing for HIV and STIs. If you have acquired an STI other than HIV, you will be offered medicine to treat it or provided information for where you may receive treatment, and study staff will discuss options available for counseling and treatment of your partner(s). This treatment or referral for treatment is available to you even if you do not enroll in this study.
- If you acquire HIV, you will need to receive care from your own health care provider or we will provide you with a referral. This study does not provide medication for treatment of HIV/AIDS.
- You will receive free male condoms, if you need them.
- You may receive free lubricant per local standard of care.

Will the study products prevent HIV infection?

The study products used in this study are placebos and do not contain any active drugs, so they will not prevent HIV. There are other known effective ways to reduce your risk of contracting HIV: the use of condoms and/or the use of oral pre-exposure prophylaxis (PrEP) or post-exposure prophylaxis (PEP) medication. Truvada® as a PrEP is an HIV prevention method in which people who do not have HIV take an oral tablet to reduce their risk of acquiring the virus. Study staff can provide you with additional information about PrEP and PEP if you are interested in learning more. If you are interested in these alternative options you may also want to discuss them with your doctor.

What if there is new information learned during this study?

We will tell you about new information from this or other studies that may affect your willingness to stay in this study. You will also be told when study results may be available, and how to learn about them.

A description of this clinical trial will be available on http://www.ClinicalTrials.gov, as required by the NIH Policy on Dissemination of NIH-Funded Clinical Trial Information. This web site will not include information that can identify you. At most, the web site will include a summary of the results. You can search this web site at any time.

Is it possible that I may be taken out of the study without my consent?

A study clinician may need to remove you from the study early without your permission if:

- The study is cancelled by the US NIH, the US Office for Human Research Protections (OHRP), MTN, government or regulatory agencies, or Institutional Review Boards/Independent Ethics Committees (IRB/IEC). An IRB/IEC is a committee that watches over the safety and rights of research participants.
- The Study Monitoring Committee (SMC) recommends that the study be stopped early. A SMC reviews the progress of the study and the kinds of effects that people report while they are participating in the study.
- You acquire HIV (see “If You Acquire HIV” section below).

- (***For individuals who can get pregnant***) You become pregnant (see "If You Become Pregnant" section below).
- You report the use of non-study rectal medications or products, except personal lubricants and usual pre-RAI douches that do not contain N-9.
- A study clinician decides that using the study products would be harmful to you, for example, you have a bad reaction to the study product.
- Other reasons that may prevent you from completing the study successfully, such as you are not able or willing to reliably keep appointments or follow study rules.

In the event that you are removed from or choose to leave this study for any reason, you will be asked to complete some of the procedures described for the Final/Early Termination Visit, if you are willing to do so.

The study clinician will ask you to stop using the study products but continue to come in for follow-up visits and procedures if you have a bad reaction to the products.

If You Acquire HIV
Your participation in this study will not cause HIV infection. However, there is always a chance that through sexual activity or other activities you may acquire HIV. In the unlikely event that you acquire HIV, study staff will give you counseling and refer you to available medical care and other services you may need. The study does not pay for this care. Tests may be performed that will allow your doctor to know what HIV drugs would be best for the treatment of your type of HIV. If the HIV test shows that you have acquired HIV, you will stop using the study products. You may be referred to other research studies. Continued study participation would be of no added benefit to you, so your participation in the study will be discontinued.

(For individuals who can get pregnant) If You Become Pregnant
The study products are not birth control methods and will not prevent pregnancy. You must use an effective method of contraception for at least 30 days (inclusive) prior to Enrollment and for the duration of study participation. You must agree to use an effective method of birth control such as birth control pills or another hormonal-based method, or an intrauterine device (IUD) inserted at least 30 days prior to enrollment, or abstain from penile-vaginal intercourse for 90 days prior to enrollment and for the duration of study participation, unless you or your partner have been sterilized (i.e., no longer able to become pregnant), and/or you only have sex that cannot lead to pregnancy (no penile-vaginal intercourse).

We do not know what effect the placebo study products may have on pregnancy, including the effect on the fetuses of individuals who use the products when pregnant. Because of this, individuals who are pregnant may not join this study. Individuals who can get pregnant and who join this study will have pregnancy tests while in the study.

If you become pregnant during the study, study staff will refer you to available medical care and other services you or your baby may need. The study does not pay for this care. You will stop using the study products and you will exit the study.

Will there be any payments if I take part in this research study?
[SITES TO INSERT INFORMATION ABOUT LOCAL REIMBURSEMENT:] You will receive [***SITES TO INSERT AMOUNT* $xx**] for your time, effort, and travel to and from the clinic at each scheduled visit. You may receive [***SITES TO INSERT AMOUNT* $xx**] for any visits which occur in between your normally scheduled visits. For responding to text messages, you will receive up

to [***SITES TO INSERT AMOUNT* $xx**]. If you are selected and agree to take part in the in-depth phone interview, you will receive [***SITES TO INSERT AMOUNT* $xx**].

What are the costs?
[SITES TO COMPLETE ACCORDING TO SITE CAPACITY] There is no cost to you for study related visits, physical/rectal exams, laboratory tests or other procedures. Treatments available to you from the study site for infections passed through sex will be given to you free of charge or you will be referred for available treatment for the duration of the study.

Are there any other studies if you cannot join this one?
There may be other studies going on at this study clinic or in the community for which you may be eligible. If you wish, we will tell you about other studies that we know about. There may also be other places where you can go for HIV counseling and testing. We will tell you about those places if you wish. If you choose not to take part in this study, it will have no effect on the regular medical care that is available to you at this clinic or elsewhere.

Who will know about my participation in this research study?
Any information about you obtained from this research will be kept as private as possible. All records related to your involvement in this research study will be kept in a [***SITES TO INSERT***]. Your identity on these records will be indicated by a number rather than by your name, and the information linking these numbers with your name will be kept separate from the research records.

Efforts will be made to keep your information confidential. However, it is not possible to guarantee confidentiality. Your personal information may be disclosed if required by law. Any publication of this study will not use your name or identify you personally. The study staff may use your personal information to verify that you are not in any other research studies. This includes studies conducted by other researchers that study staff know about.

Your records may be reviewed by:
- Study staff
- Site IRBs/IECs
- CONRAD, the organization that supplies the placebo insert
- Representatives of the US Federal Government, including OHRP, NIH and/or contractors of NIH
- Other local, US or international regulatory authorities

*[**Sites to include/amend the following:] [LOCAL/STATE/NATIONAL**]* regulations require study staff to report the names of people who test positive for HIV and other infections passed during sex to the *[**LOCAL HEALTH AUTHORITY**]*. Outreach workers from the *[**LOCAL HEALTH AUTHORITY**]* may then contact you about informing your partners, since they also should be tested. If you do not want to inform your partners yourself, the outreach workers will contact them, according to the confidentiality guidelines of the *[**HEALTH AUTHORITY**].*

[Sites to include/amend the following:]
The researchers will do everything they can to protect your privacy. In addition to the efforts of the study staff to help keep your personal information private, we have obtained a Certificate of Confidentiality from the US Federal Government. This Certificate protects study staff located in the US from being forced to tell people who are not connected with this study, such as the court system, about your participation or information you give for study purposes. However, if the study staff learns of possible child abuse and/or neglect or a risk of harm to you or others, they will be

required to tell the proper authorities. This Certificate does not prevent you from releasing information about yourself and your participation in the study.

What if I am injured as a result of participating in this study?
*[**SITES TO SPECIFY INSTITUTIONAL POLICY**:]* It is unlikely that you will be injured as a result of study participation. If you are injured, the *[**institution**]* will give you immediate necessary treatment for your injuries. You *[**will/will not**]* have to pay for this treatment. You will be told where you can receive additional treatment for your injuries. The U.S. National Institutes of Health (NIH) does not have a mechanism to pay money or give other forms of compensation for research related injuries. You do not give up any legal rights by signing this consent form.

May I withdraw my consent for participation in this research study?
*[**SITE TO SPECIFY INSTITUTIONAL POLICY**:]* Taking part in this study is completely voluntary. You may choose not to take part in this study or leave this study at any time. If you choose not to participate or to leave the study, you will not lose the benefit of services to which you would otherwise be entitled at this clinic, nor will the confidentiality of the care provided for you here be affected. You should feel free coming back to this facility even if you decide not to participate in this study. If you want the results of the study after the study is over, let the study staff members know.

What do I do if I have questions?
If you ever have any questions about the study, or if you have a research-related injury, you should contact *[**insert name of the investigator or other study staff]*** at ***[insert telephone number and/or physical address**]*.

If you have questions about your rights as a research participant, you should contact **[insert name or title of person on the IRB or other organization appropriate for the site]** at **[insert physical address and telephone number]**.

SIGNATURES- VOLUNTARY CONSENT

[INSERT SIGNATURE BLOCKS AS REQUIRED BY THE LOCAL IRB/IEC]: If you have read this consent form, or had it read and explained to you, and you understand the information, and you voluntarily agree to the study, please sign your name or make your mark below.

______________________	____________________________	________________
Participant Name (print)	Participant Signature/Mark	Date

______________________	____________________________	________________
Study Staff Conducting Consent Discussion (print)	Study Staff Signature	Date

______________________	____________________________	________________
Witness Name (print)	Witness Signature	Date

REFERENCES

1. McGowan I. Microbicides for HIV prevention: Reality or hope? Curr Opin Infect Dis 2010;23:26-31.
2. Leynaert B, Downs AM, de V, I. Heterosexual transmission of human immunodeficiency virus: variability of infectivity throughout the course of infection. European Study Group on Heterosexual Transmission of HIV. Am J Epidemiol 1998;148:88-96.
3. Vittinghoff E, Douglas J, Judson F, McKirnan D, MacQueen K, Buchbinder SP. Per-contact risk of human immunodeficiency virus transmission between male sexual partners. Am J Epidemiol 1999;150:306-11.
4. Gorbach PM, Manhart LE, Hess KL, Stoner BP, Martin DH, Holmes KK. Anal intercourse among young heterosexuals in three sexually transmitted disease clinics in the United States. Sex Transm Dis 2009;36:193-8.
5. Carlos JA, Bingham TA, Stueve A, et al. The role of peer support on condom use among Black and Latino MSM in three urban areas. AIDS Educ Prev 2010;22:430-44.
6. Beyrer C, Baral SD, van Griensven F, et al. Global epidemiology of HIV infection in men who have sex with men. Lancet 2012;380:367-77.
7. Sullivan PS, Hamouda O, Delpech V, et al. Reemergence of the HIV epidemic among men who have sex with men in North America, Western Europe, and Australia, 1996-2005. Ann Epidemiol 2009;19:423-31.
8. WHO. HIV/AIDS Surveillance in Europe 2017 - 2016 data. The European Centre for Disease Prevention and Control (ECDC) and the WHO Regional Office for Europe; 2017.
9. HIV among gay and bisexual men. CDC, 2018. (Accessed April 4, 2018, at https://www.cdc.gov/hiv/group/msm/index.html.)
10. Stall R, Duran L, Wisniewski SR, et al. Running in Place: Implications of HIV Incidence Estimates among Urban Men Who Have Sex with Men in the United States and Other Industrialized Countries. AIDS Behav 2009;13:615-29.
11. Millett GA, Jeffries WLt, Peterson JL, et al. Common roots: a contextual review of HIV epidemics in black men who have sex with men across the African diaspora. Lancet 2012;380:411-23.
12. HIV prevalence among South African MSM twice as high as general population. 2009. (Accessed October 26, 2017, at http://www.aidsmap.com/HIV-prevalence-among-South-African-MSM-twice-as-high-as-general-population/page/1323795/.)
13. Baral SD, Poteat T, Stromdahl S, Wirtz AL, Guadamuz TE, Beyrer C. Worldwide burden of HIV in transgender women: a systematic review and meta-analysis. Lancet Infect Dis 2013;13:214-22.
14. Clark H, Babu AS, Wiewel EW, Opoku J, Crepaz N. Diagnosed HIV Infection in Transgender Adults and Adolescents: Results from the National HIV Surveillance System, 2009–2014. AIDS Behav 2017;21:2774-83.
15. Herbst JH, Jacobs ED, Finlayson TJ, McKleroy VS, Neumann MS, Crepaz N. Estimating HIV prevalence and risk behaviors of transgender persons in the United States: a systematic review. AIDS Behav 2008;12:1-17.
16. Operario D, Soma T, Underhill K. Sex work and HIV status among transgender women: systematic review and meta-analysis. J Acquir Immune Defic Syndr 2008;48:97-103.

17. Organization WH. Consolidated guidelines on HIV prevention, diagnosis, treatment and care for key populations. In: HIV/AIDS Do, ed. Geneva, Switzerland2014.
18. Schmidt A, Benvenuti S, Breveglieri M, et al ftEc. The European MSM internet survey (EMIS) UNGASS indicators2011.
19. Abell N, Rutledge SE, McCann TJ, Padmore J. Examining HIV/AIDS provider stigma: assessing regional concerns in the islands of the Eastern Caribbean. AIDS Care 2007;19:242-7.
20. Vu BN, Girault P, Do BV, Colby D, Tran LT. Male sexuality in Vietnam: the case of male-to-male sex. Sex Health 2008;5:83-8.
21. Adam PC, de Wit JB, Toskin I, et al. Estimating levels of HIV testing, HIV prevention coverage, HIV knowledge, and condom use among men who have sex with men (MSM) in low-income and middle-income countries. J Acquir Immune Defic Syndr 2009;52 Suppl 2:S143-51.
22. Blair JM, Fleming PL, Karon JM. Trends in AIDS incidence and survival among racial/ethnic minority men who have sex with men, United States, 1990-1999. J Acquir Immune Defic Syndr 2002;31:339-47.
23. Sandfort TGM, Nel J, Rich E, Reddy V, Yi H. HIV testing and self-reported HIV status in South African MSM: Results from a community-based survey. Sex Transm Infect 2008;84:425-9.
24. Hall HI, Byers RH, Ling Q, Espinoza L. Racial/ethnic and age disparities in HIV prevalence and disease progression among men who have sex with men in the United States. Am J Public Health 2007;97:1060-6.
25. AIDS By the Numbers-2016. 2016. (Accessed 10/21/2016, 2016, at http://www.unaids.org/en/resources/documents/2016/AIDS-by-the-numbers.)
26. Weissman A, Ngak S, Srean C, Sansothy N, Mills S, Ferradini L. HIV Prevalence and Risks Associated with HIV Infection among Transgender Individuals in Cambodia. PLoS One 2016;11:e0152906.
27. Chemnasiri T, Netwong T, Visarutratana S, et al. Inconsistent condom use among young men who have sex with men, male sex workers, and transgenders in Thailand. AIDS Educ Prev 2010;22:100-9.
28. CDC. HIV testing and risk behaviors among gay, bisexual, and other men who have sex with men – United States2013.
29. Casalino E, Choquet C, Leleu A, et al. Trends in condom use and risk behaviours after sexual exposure to HIV: a seven-year observational study. PLoS One 2014;9:e104350.
30. Elford J. Changing patterns of sexual behaviour in the era of highly active antiretroviral therapy. Curr Opin Infect Dis 2006;19:26-32.
31. Elford J, Bolding G, Davis M, Sherr L, Hart G. Trends in sexual behaviour among London homosexual men 1998-2003: implications for HIV prevention and sexual health promotion. Sex Transm Infect 2004;80:451-4.
32. Phillips AN, Cambiano V, Nakagawa F, et al. Increased HIV incidence in men who have sex with men despite high levels of ART-induced viral suppression: analysis of an extensively documented epidemic. PLoS One 2013;8:e55312.
33. Pines HA, Gorbach PM, Weiss RE, et al. Acceptability of potential rectal microbicide delivery systems for HIV prevention: a randomized crossover trial. AIDS Behav 2013;17:1002-15.

34. Paz-Bailey G, Mendoza MC, Finlayson T, et al. Trends in condom use among MSM in the United States: the role of antiretroviral therapy and seroadaptive strategies. AIDS 2016;30:1985-90.
35. Grant RM, Anderson PL, McMahan V, et al. Uptake of pre-exposure prophylaxis, sexual practices, and HIV incidence in men and transgender women who have sex with men: A cohort study. Lancet Infect Dis 2014;14:820-9.
36. Reece M, Herbenick D, Schick V, Sanders SA, Dodge B, Fortenberry JD. Condom Use Rates in a National Probability Sample of Males and Females Ages 14 to 94 in the United States. The Journal of Sexual Medicine 2010;7:266-76.
37. Herbenick D, Reece M, Schick V, Sanders SA, Dodge B, Fortenberry JD. Sexual Behavior in the United States: Results from a National Probability Sample of Men and Women Ages 14–94. The Journal of Sexual Medicine 2010;7:255-65.
38. Rosenberger JG, Reece M, Schick V, et al. Sexual Behaviors and Situational Characteristics of Most Recent Male-Partnered Sexual Event among Gay and Bisexually Identified Men in the United States. The Journal of Sexual Medicine 2011;8:3040-50.
39. Fonner VA, Dalglish SL, Kennedy CE, et al. Effectiveness and safety of oral HIV preexposure prophylaxis for all populations. AIDS 2016;30:1973-83.
40. Weber J, Tatoud R, Fidler S. Postexposure prophylaxis, preexposure prophylaxis or universal test and treat: the strategic use of antiretroviral drugs to prevent HIV acquisition and transmission. AIDS 2010;24 Suppl 4:S27-39.
41. Liu AY, Vittinghoff E, Chillag K, et al. Sexual risk behavior among HIV-uninfected men who have sex with men participating in a tenofovir preexposure prophylaxis randomized trial in the United States. J Acquir Immune Defic Syndr 2013;64:87-94.
42. Callahan R, Nanda K, Kapiga S, et al. Pregnancy and contraceptive use among women participating in the FEM-PrEP trial. J Acquir Immune Defic Syndr 2015;68:196-203.
43. Murnane PM, Heffron R, Ronald A, et al. Pre-exposure prophylaxis for HIV-1 prevention does not diminish the pregnancy prevention effectiveness of hormonal contraception. AIDS 2014;28:1825-30.
44. Sullivan PS, Carballo-Dieguez A, Coates T, et al. Successes and challenges of HIV prevention in men who have sex with men. Lancet 2012;380:388-99.
45. Krakower D, Mayer KH. Engaging healthcare providers to implement HIV pre-exposure prophylaxis. Curr Opin HIV AIDS 2012;7:593-9.
46. Zablotska IB, Prestage G, de Wit J, Grulich AE, Mao L, Holt M. The informal use of antiretrovirals for preexposure prophylaxis of HIV infection among gay men in Australia. J Acquir Immune Defic Syndr 2013;62:334-8.
47. Mimiaga MJ, White JM, Krakower DS, Biello KB, Mayer KH. Suboptimal awareness and comprehension of published preexposure prophylaxis efficacy results among physicians in Massachusetts. AIDS Care 2014;26:684-93.
48. Gilead Sciences. TRUVADA (emtricitabine and tenofovir disoproxil fumarate) tablets: Packge Insert (2016). 2016.
49. Garg S, Goldman D, Krumme M, Rohan LC, Smoot S, Friend DR. Advances in development, scale-up and manufacturing of microbicide gels, films, and tablets. Antiviral Res 2010;88:S19-S29.
50. Rosenberg ZF, Devlin B. Future strategies in microbicide development. Best Practice & Research Clinical Obstetrics & Gynaecology 2012;26:503-13.

51. van der Straten A, Van DL, Haberer JE, Bangsberg DR. Unraveling the divergent results of pre-exposure prophylaxis trials for HIV prevention. AIDS 2012;26:F13-F9.
52. Baeten JM, Haberer JE, Liu AY, Sista N. Pre-exposure prophylaxis for HIV prevention: where have we been and where are we going? Journal of acquired immune deficiency syndromes (1999) 2013;63:S122-S9.
53. Brown E, Palanee-Philips T, Marzinke M, et al. Residual dapivirine ring levels indicate higher adherence to vaginal ring is associated with HIV-1 protection. AIDS 2016, July 18-22, 2016, Durban, South Africa 2016:Abstract # TUAC0105LB.
54. Ventuneac A, Carballo-Dieguez A, McGowan I, et al. Acceptability of UC781 gel as a rectal microbicide among HIV-uninfected women and men. AIDS Behav 2010;14:618-28.
55. Carballo-Dieguez A, Dolezal C, Bauermeister JA, O'Brien W, Ventuneac A, Mayer K. Preference for gel over suppository as delivery vehicle for a rectal microbicide: results of a randomised, crossover acceptability trial among men who have sex with men. Sex Transm Infect 2008;84:483-7.
56. Carballo-Dieguez A, Giguere R, Dolezal C, et al. Rectal-specific microbicide applicator: evaluation and comparison with a vaginal applicator used rectally. AIDS Behav 2014;18:1734-45.
57. Javanbakht M, Stahlman S, Pickett J, LeBlanc MA, Gorbach PM. Prevalence and types of rectal douches used for anal intercourse: results from an international survey. BMC Infect Dis 2014;14:95.
58. Carballo-Dieguez A, Bauermeister JA, Ventuneac A, Dolezal C, Balan I, Remien RH. The use of rectal douches among HIV-uninfected and infected men who have unprotected receptive anal intercourse: implications for rectal microbicides. AIDS Behav 2008;12:860-6.
59. CONRAD. A 14-Day Rectal Irritation and Pharmakinetic Study of Elvitegravir in Rabbits - Draft Pathology Report (MPI Study Number: 1645-114). 2017.
60. Meisel JL, Bergman D, Graney D, Saunders DR, Rubin CE. Human rectal mucosa: proctoscopic and morphological changes caused by laxatives. Gastroenterology 1977;72.
61. Leyva FJ, Bakshi RP, Fuchs EJ, et al. Isoosmolar Enemas Demonstrate Preferential Gastrointestinal Distribution, Safety, and Acceptability Compared with Hyperosmolar and Hypoosmolar Enemas as a Potential Delivery Vehicle for Rectal Microbicides. AIDS Res Hum Retroviruses 2013;29:1487-95.
62. Yerkes EB, Rink RC, King S, Cain MP, Kaefer M, Casale AJ. Tap water and the Malone antegrade continence enema: a safe combination? The Journal of urology 2001;166:1476-8.
63. Chemical IO, Kuznik J. Witepsol® H5-based suppository products currently on the market. In: Rohan L, ed.2017.
64. Stahlman S, Gorbach P. Rectal behaviors: a global overview. Microbicides 2012. Sydney2012.
65. Chakrapani V, Newman PA, Shunmugam M, et al. "Like Holding an Umbrella Before It Rains": Acceptability of Future Rectal Microbicides Among Men Who Have Sex With Men in India-A Modified Technology Acceptance Model. Qual Health Res 2017;27:1236-48.
66. Newman PA, Cameron MP, Roungprakhon S, Tepjan S, Scarpa R. Acceptability and Preferences for Hypothetical Rectal Microbicides among a Community Sample of Young

Men Who Have Sex with Men and Transgender Women in Thailand: A Discrete Choice Experiment. AIDS Behav 2016;20:2588-601.
67. Zhang G, Zhang H, Yu H, et al. Hypothetical Rectal Microbicide Acceptability and Factors Influencing It among Men Who Have Sex with Men in Tianjin, China. PLoS One 2016;11:e0156561.
68. Brown J, Haines S, Wilding IR. Colonic spread of three rectally administered mesalazine (Pentasa) dosage forms in healthy volunteers as assessed by gamma scintigraphy. Aliment Pharmacol Ther 1997;11:685-91.
69. Galea JT, Kinsler JJ, Imrie J, Nurena CR, Sanchez J, Cunningham WE. Rectal douching and implications for rectal microbicides among populations vulnerable to HIV in South America: a qualitative study. Sex Transm Infect 2014;90:33-5.
70. Noor SW, Rosser BR. Enema use among men who have sex with men: a behavioral epidemiologic study with implications for HIV/STI prevention. Arch Sex Behav 2014;43:755-69.
71. Galea JT, Kinsler JJ, McLean S, et al. Rectal Douching Prevalence and Practices Among Peruvian Men Who have Sex with Men and Transwomen: Implications for Rectal Microbicides. AIDS Behav 2016;20:2555-64.
72. Lamblet LCR, da Silva RJC. Prevalence and types of rectal douches used for anal intercourse among men who have sex with men in Brazil. BMJ Open 2017;7:e011122.
73. Mitchell JW, Sophus AI, Lee JY, Petroll AE. Anal Douche Practices and Willingness to Use a Rectal Microbicide Enema for HIV Prevention and Associated Factors Among an Internet Sample of HIV-Negative and HIV-Discordant Male Couples in the US. AIDS Behav 2016;20:2578-87.
74. Hambrick HR, Park SH, Goedel WC, et al. Rectal Douching Among Men Who Have Sex with Men in Paris: Implications for HIV/STI Risk Behaviors and Rectal Microbicide Development. AIDS Behav 2017.
75. Javanbakht M, Murphy R, Gorbach P, LeBlanc M-A, Pickett J. Preference and practices relating to lubricant use during anal intercourse: implications for rectal microbicides. Sexual Health 2010;7:193-8.
76. Carballo-Dieguez A, Stein Z, Saez H, Dolezal C, Nieves-Rosa L, Diaz F. Frequent use of lubricants for anal sex among men who have sex with men: the HIV prevention potential of a microbicidal gel. Am J Public Health 2000;90:1117-21.
77. Frasca T, Giguere R, Ibitoye M, et al. Lessons for Rectal Microbicide Development From an Acceptability Trial of a Placebo Gel Applied Prior to Receptive Anal Intercourse. Arch Sex Behav 2017;46:1101-9.
78. Stone E, Heagerty P, Vittinghoff E, et al. Correlates of condom failure in a sexually active cohort of men who have sex with men. J Acquir Immune Defic Syndr Hum Retrovirol 1999;20:495-501.
79. Baral S, Trapence G, Motimedi F, et al. HIV prevalence, risks for HIV infection, and human rights among men who have sex with men (MSM) in Malawi, Namibia, and Botswana. PLoS One 2009;4:e4997.
80. Gorbach PM, Weiss RE, Fuchs E, et al. The slippery slope: lubricant use and rectal s exually transmitted infections: a newly identified risk. Sex Transm Dis 2012;39:59-64.
81. Carballo-Dieguez A, Bauermeister J, Ventuneac A, Dolezal C, Mayer K. Why rectal douches may be acceptable rectal-microbicide delivery vehicles for men who have sex with men. Sex Transm Dis 2010;37:228-33.
