# Supplementary material for: A randomized trial of safety, acceptability and adherence of three rectal microbicide placebo formulations among young sexual and gender minorities who engage in receptive anal intercourse (MTN-035)
Source: PLoS One. 2023 Apr 12;18(4):e0284339. doi: 10.1371/journal.pone.0284339 (PMC10096248; doi:10.1371/journal.pone.0284339)
Supplement: Attachment — Submitted filename: Response to Reviewers.docx [file pone.0284339.s003.docx]

PONE-D-22-27511R1

A Randomized Trial of Safety, Acceptability and Adherence of Three Rectal Microbicide Placebo Formulations among Young Sexual and Gender Minorities Who Engage in Receptive Anal Intercourse (MTN-035)

PLOS ONE

Thank you for the opportunity to revise our manuscript under consideration in PLOS ONE. We have addressed the remaining questions/comments from the Reviewers, and uploaded both a version with track changes and an unmarked version of the manuscript with the requested revisions.

**Reviewer #2:**

The authors appear to have responded to reviewers’ comments satisfactorily, although my comments have been rearranged and paraphrased so it was difficult to keep track of authors’ responses.

Table 4 “Odds Ratio and 95% Confidence Interval” row looks like it's the percentage with high acceptability with missing data removed, with 95%CI i.e. not an odds ratio. I strongly agree that acceptability 95%CI should be quoted, especially now the authors have made it clear that 70% acceptability is an important benchmark. However, it seems odd to give the % Yes at the top of that table, which includes those with missing data in the denominator, then a few rows down present a very slightly different estimate (because missing data are removed). So the table needs correct labelling and clearer signposting as to which statistic (with or without those with missing data) is the most important outcome. Maybe just state number with missing data as a footnote and present the percentages excluding missing data only.

- Thank you. We have removed the mislabeling in our row stating Odds Ratio and now clarify that it’s the percent of participants with high acceptability/adherence.
- We have deleted the recommended rows noting missingness and added a Note instead that states:
  - Notes. Estimates exclude missing data for acceptability (Rectal insert (n=3); Suppository (n=2); Enema (n=5)) and adherence (Rectal insert (n=3); Suppository (n=3); Enema (n=2)).

**Reviewer #3:**

All through the article, beginning with the abstract and all over the results, please indicate the confidence intervals reported. This can be as simple as changing 'CI' to '95%CI' if indeed these are 95% confidence intervals.

- Thank you. We have now edited the text to document that we are referring to 95% Confidence Intervals.
